# Terroir Dynamics: Impact of Vineyard and Canopy Treatment with Chitosan on Anthocyanins, Phenolics, and Volatile and Sensory Profiles of Pinot Noir Wines from South Tyrol

**DOI:** 10.3390/molecules29091916

**Published:** 2024-04-23

**Authors:** Prudence Fleur Tchouakeu Betnga, Simone Poggesi, Aakriti Darnal, Edoardo Longo, Elena Rudari, Emanuele Boselli

**Affiliations:** 1 Faculty of Agricultural, Environmental and Food Sciences, Free University of Bozen-Bolzano, Piazza Università 1, 39100 Bolzano, Italy; prudencefleur.tchouakeubetnga@unibz.it (P.F.T.B.); aakriti.darnal@student.unibz.it (A.D.); elena.rudari@gmail.com (E.R.); emanuele.boselli@unibz.it (E.B.); 2 Oenolab, NOITechPark Alto Adige/Südtirol, Via A. Volta 13B, 39100 Bolzano, Italy; 3 Food Experience and Sensory Testing (Feast) Lab., Massey University, Palmerston North 4410, New Zealand; s.poggesi@massey.ac.nz

**Keywords:** pinot noir wines, chemical fingerprint, sensory profile, enological parameters, vineyard location, chitosan treatment, chromatography, mass spectrometry

## Abstract

The effects of canopy treatment with chitosan and the effects of the vineyard location on the quality parameters, volatile and non-volatile profiles, and sensory profile of Pinot Noir wines from South Tyrol (Italy) were studied. Multivariate statistical analysis was applied to identify the most relevant compounds associated with the variability in phenolics and anthocyanins (analyzed by UHPLC-MS), volatile components (HS-SPME-GCxGC-ToF/MS), and basic enological parameters. A clear separation of low-altitude wines (350 m.a.s.l.), which had a high concentration of most of the identified volatile compounds, compared to high-altitude wines (800 and 1050–1150 m.a.s.l.) was pointed out. Low altitude minimized the concentration of the most significant anthocyanins in wines from a valley bottom, presumably due to reduced sun exposure. Wines obtained from chitosan-treated canopies, and, more particularly, those subjected to multiple treatments per year showed a higher amount of the main non-volatile phenolics and were sensorially described as having “unpleasant flavors” and “odors”, which might suggest that grape metabolism is slightly altered compared to untreated grapevines. Thus, optimization of the treatment with chitosan should be further investigated.

## 1. Introduction

Thanks to its soil, geographical location in the middle of the Alpine mountain range system, and climatic conditions, South Tyrol offers favorable conditions for a successful cultivation of a wide range of grape varieties, also considering the altitude range (vineyards are found from 200 up to 1100–1300 m.a.s.l.). However, South Tyrol is one of the smallest winegrowing regions in Italy, with a total viticultural area of around 5480 ha out of 718,000 hectares in Italy, of which about 479 are for Pinot Noir (the third most cultivated red grape variety), equivalent to 8.7% of the total provincial viticultural area [1,2]. The cultivation of Pinot Noir was started almost two centuries ago by the Archduke Johann of Habsburg (1782–1859) [3]. Unlike other viticultural areas, about 99.4% of Pinot Noir is destined to produce DOC wines. Pinot Noir wines produced from the vineyards located in Mazzon (a village in the municipality of Egna) are known nationally and internationally and received great recognition in several competitions [4].

Pinot Noir is a grape variety showing fast ripening in hot climates, while cold temperatures and high humidity levels during the flowering period are responsible for millerandage and shedding. Also, clay and humid soils are unsuitable for this variety [5]. Pinot Noir has low fertility and produces low quantities of grapes per vine. The skins are rich in polyphenols and the berries have a moderate acidity and a good sugar content [6]. In the presence of calcareous soils and a cold environment, Pinot Noir gives the best results in terms of complexity, refinement, and aromatic characteristics. The red wines produced from this variety have a light color and great aging potential in barrels [7]. Merkyté et al. [8] have demonstrated that Pinot Noir from South Tyrol is rich in cyclic and non-cyclic B-type proanthocyanidins, which are powerful chemical markers suitable to characterize these wines.

To harvest quality grapes, it is important to manage gray mold, leafhoppers, and downy mildew on the canopy. Fungicides are often used for this purpose, but due to safety concerns [9], natural compounds for canopy treatment, such as chitosan, are increasingly taken into consideration. Chitosan is a natural polymer approved for the treatment of organic vineyards based on its fungicide effect. Besides the protection it offers, chitosan also has the potential to modulate the polyphenolic content in black grapes, hence improving the color of the resulting red wines [10]. Chitosan is a polymer of β-1,4-D-glucosamine; it is a polysaccharide obtained after partial deacetylation of chitin (from 15% to 90%), the widespread natural polymer constituting the exoskeleton of crustaceans and the cuticles of insects. High percentages of chitin are also present in the cell walls of some fungi, including vine pathogens. The use of chitosan on the canopy vines does not influence the productive–vegetative parameters (yield of the plant, weight of the bunch, Ravaz index) and the growth and ripening of the berries [11]. The resistance to fungi induced by chitosan in plants leads to an increase in the content of polyphenolic phytoalexins due to the stimulation of the phenylpropanoid pathway [12]. The changes in berry composition induced by chitosan treatments affect the chemical properties of wines. The frequency of use of foliar treatments with chitosan performed regularly from spring to harvest can be increased compared to the use of conventional fungicides. This seems to lead to an increase in the total polyphenol content of grapes and wine as well as their antioxidant activity [12]. Research performed by Tessarin et al. [11] has shown that three treatments (at the beginning, at the end of veraison, and pre-harvest) with chitosan cause significant differences in the quantities of (+)-catechin, (−)-epicatechin, hydroxycinnamic acids, ferulic acid, coutaric acid, and procyanidin B2 in wines from Cabernet Sauvignon grapes and no effect on Sangiovese grapes. Several researchers have demonstrated that canopy treatment with chitosan alone or in combination with other phytochemicals induced a differentiated response in the phenolic composition in grapes [13,14], in cvs. Cabernet Sauvignon [11,15], Montepulciano [12], Groppello, Merlot [16], Tempranillo [17], Tinto Cão, Touriga Franca [10,18], Fetească neagră [13], and Sousão [19]. Recently, Artem et al. [13] have shown that the content of anthocyanins and tannins in grape skins increases with the total dose of chitosan applied in the vineyard and that the color intensity of wines increased as well as phenolic compounds such as gallic acid, catechin, epicatechin, and *trans*-resveratrol.

According to Portu et al. [17], the use of two treatments with chitosan (one at veraison and the other a week after it) on the canopy barely influenced the phenolic content of the grape or wine (Vitis vinifera L. Tempranillo). No significant differences in the total content of flavonols, flavanols, and non-flavonoids between the control and the treatments for both grapes and wines were observed. Some scientific studies also highlighted that only a pre-harvest application of chitosan may not affect the total phenolic and anthocyanin content of grape and related wine of Cabernet Sauvignon [15]. Regarding the sensory profile, some researchers have performed studies on wines obtained from canopy treated with chitosan and most results have shown positive sensory notes. For instance, Vitalini et al. [20] demonstrated that the application of chitosan in the vineyard helps in improving the volatile profile, flavor, and taste of Groppello wine.

In the same perspective as the available literature, this current research aims to determine the effect of agronomic management (chitosan treatment on the canopy) as well as the effect of vineyard location and sun exposure on the volatile, non-volatile, and sensory profiles of South Tyrolean Pinot Noir wines.

## 2. Results and Discussion

The chemical and sensory datasets were analyzed to check for the influence of the vineyards (Eggerhof, Patone, Aldino, and Mazzon) and the treatment on the canopy (control, chitosan applied only once a year before harvest, and chitosan applied three times per year at the beginning of flowering, end of veraison, and pre-harvest) as factors. To analyze more homogeneous datasets, two separate data analyses were considered, firstly to describe the effect of vineyard and secondly the impact of the treatment of the canopy with chitosan on the wine. For each entry, the following points are reported and discussed: MFA loadings and score plot of the basic enological parameters, phenolics, anthocyanins, volatile compounds, and sensory attributes. Regression models were also constructed by combining the data for wines obtained from the four vineyards, and finally, an ANOVA test for the different variables was presented. The volatile compounds identified in wines are presented in Table S1.

### 2.1. Multiple Factor Analysis (MFA)

#### 2.1.1. Effect of Vineyard Location

**Figure 1:**
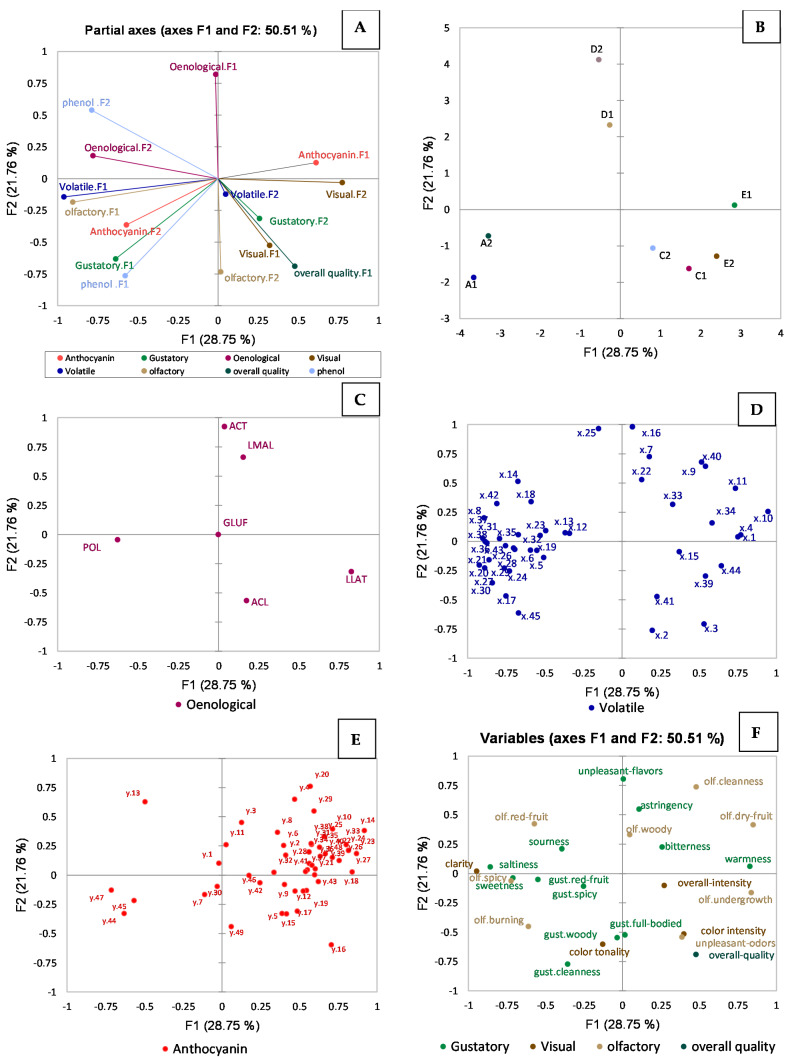
MFA of wines from the four different vineyards. (**A**) shows the projection of the Principal Components of the different datasets applied on the first two MFA partial axes, (**B**) represents the observation plot, and (**C**–**F**) show the basic enological variables, volatile compounds, anthocyanins, and sensory analysis variables, respectively. The identified volatile compounds in (**D**) are presented in Table S1. Identified anthocyanin compounds are presented in Figure S1 (Supplementary Materials File S1). In (**B**), the symbol (**A**) means Mazzon vineyard, (**C**) Aldino vineyard, (**D**) Patone vineyard, and (**E**) Eggerhof vineyard. ACL: acetic acid (g/L); LLAT: lactic acid (g/L); LMAL: malic acid (g/L); ACT: tartaric acid (g/L); GLUF: glucose–fructose (g/L), and POL: total polyphenol (mg/L).

The effect of vineyard location was evaluated, and Figure 1 (A: projection of the dataset components on the MFA partial axes; B: score plot; C–F: projection of the loadings separated by individual datasets) represents the MFA model of the wines including (as separate datasets) the basic enological parameters, sensory analysis results, and volatile compounds, non-volatile phenolics, and anthocyanins. Assignment of the volatile compounds is presented in the Supporting Information File S1 (according to comparison of the observed retention times and spectra with spectral databases and LRI references [21,22,23,24,25,26,27,28,29,30,31,32,33,34,35,36,37]). This approach was used to bring together the different datasets into a single multivariate representation to obtain an overview of correlations between variables and trends between observations. The first two partial axes accounted for 50.51% of the total variance. The first partial axis, F1, represents 28.75%, while F2 represents 21.76% of the total variability. To carry out a more accurate evaluation, the wine samples were analyzed in replicates. In Figure 1A,B, a clear separation along the F2 axis of wines from Patone (D) can be seen due to the contribution of basic enological parameters-F1 and phenolic compounds-F2. Similarly, volatile compounds-F1, olfactory attributes-F1, basic enological parameters-F2, anthocyanin compounds-F2, gustatory attributes-F1, and phenolic compounds-F1 mostly contributed to the separation of wines from the Mazzon vineyard (A). Instead, volatile compounds-F2, gustatory descriptors-F2, visual attributes-F1, olfactory attributes-F2, and the overall quality-F1 correlated with the wines from the Aldino vineyard (C). Meanwhile, the anthocyanin compounds-F1 and visual descriptors-F2 separated the wines from Eggerhof (E1).

Figure 1C represents the loading plot for the projection of basic enological parameters on the first two partial axes, showing a strong correlation of tartaric and malic acids with Patone (D) wines, while acetic acid was anti-correlated; Eggerhof (E) and Aldino (C) wines had a high concentration of lactic and acetic acids, while the total polyphenols as well as tartaric and malic acids were low in these wines. By contrast, the Mazzon wines had a high content of total polyphenols.

The projection of the volatile compounds listed in Table S1 on the first two partial axes of the MFA is presented in Figure 1D. It is possible to evidence some of the most significant volatile compounds contributing to wines from grapes harvested in the Mazzon vineyard (A): 1-octanol (x.17); ethyl benzeneacetate (x.30); 3-methylthio-1-propanol (x.27); 3-methyl-1-butanol acetate (x.5); 2-methyl-1-propanol (x.6); 2,3-butanediol (x.19); butyrolactone (x.21); α-terpineol (x.26); and citronellol (x.29).

According to the literature [38], these compounds have specific sensory notes: 2-methyl-1-propanol (x.6) is a higher alcohol (fusel alcohol) with a high or very high perception threshold at 40,000 μg/L [39], which is characterized by a warm sensation due to the alcohol. Butyrolactone (x.21) has a slightly pungent caramellic and sweet odor with fruity undertones and a bitter, mildly sweet caramellic herbaceous taste; 3-methyl-1-butanol acetate (x.5) is associated with banana/fruity notes (isoamyl acetate); α-terpineol (x.26) has a characteristic lilac odor with a sweet reminiscence of peach. Citronellol (x.29) has a typical rose-like odor.

Figure 1B,D show that Aldino (C) and Eggerhof (E) wines were grouped and separated from the others along F1, and the main volatile compounds characterizing these samples were ethyl acetate (x.2); n-propyl acetate (x.3); benzaldehyde (x.15); 2(3*H*)-furanone, 5-butyldihydro-4-methyl-, *cis*-whisky lactone (x.39); 4-ethyl phenol (x.41); ethyl hexadecanoate (x.44); acetaldehyde (x.1); 1-propanol (x.4); 2*H*-pyran-2-one, tetrahydro-3,6-dimethyl (x.10); and benzyl alcohol (x.33), and their sensory notes are presented according to [38]. Acetaldehyde has a characteristic pungent and ethereal odor, while ethyl acetate has a pleasant, ethereal, fruity, brandy-like odor reminiscent of pineapple at low concentrations and somewhat nauseating at high concentrations. n-propyl acetate has a fruity (pear–raspberry) odor with a pleasant, bittersweet flavor reminiscent of pear on dilution. 1-propanol has an alcoholic odor and a characteristic ripe, fruity flavor. Benzyl alcohol has a pleasant, characteristic fruity odor and a slightly pungent, sweet taste; the note tends to become like that of benzyl aldehyde on aging. 4-ethylphenol has a powerful woody phenolic, medicinal, and sweet odor. Benzaldehyde has a characteristic odor and aromatic taste like bitter almond, while 2(3*H*)-furanone, 5-butyldihydro-4-methyl-, (*cis*-whisky lactone) may be associated with coconut notes [40].

Figure 1B,D present a clear separation of the Patone vineyard (D) from the others and the main volatile compounds contributing to this separation were ethyl 4-decenoate (x.25); 3-methyl-1-butanol (x.7); ethyl decanoate (x.22); and ethyl 2-hydroxypropanoate (x.9). ethyl 4-decenoate has a fruity, green, ester-like, sweet, and aldehyde odor like orange citrus, while ethyl 2-hydroxypropanoate has a light, ethereal, buttery odor [38].

The differences in the volatile profile between bottles considering the vineyard location as a factor were also evaluated by one-way ANOVA. The results showing only the significant compounds with Tukey’s test (*p* < 0.05) are presented in Table 1. Considering the variables 1-propanol (x.4) and benzaldehyde (x.15), the relative abundances are more than double in the Eggerhof vineyard than in the other vineyards, while the opposite situation was observed for 3-methyl-1-pentanol (x.8); 1-octanol (x.17); butyrolactone (x.21); 3-methylthio-1-propanol (x.27); citronellol (x.29); ethyl benzeneacetate (x.30); phenylethyl alcohol (x.38); and 2,4-di-tert-butylphenol (x.45), showing a higher concentration in the Mazzon vineyard (A) compared to the other vineyards; the lowest area intensity was observed for the Eggerhof (E) vineyard. On the other hand, the volatile compounds furfural (x.14) and 4-ethyl-phenol (x.41) were the main compounds that significantly differentiated the Aldino (C) vineyard from the others. 4-ethyl-phenol showed a higher amount in samples from the Aldino vineyard, while furfural had the lowest concentration. The fact that these wines were aged in barriques for about one month before sampling indicates that these results could be much more affected by the storage in barriques than the vineyard provenance.

Figure 1E represents the loading plot for the anthocyanins projected on the first two partial axes of the MFA. Most anthocyanins correlated with Eggerhof and Aldino wines, while Patone wines were mostly differentiated by peonidin-3*O*-glucoside. The most intense peaks were identified and are presented in Figure S1 (Supplementary Materials File S1).

Regarding the sensory attributes, the loading plot from the MFA is presented in Figure 1F. Patone samples were characterized by “astringency”, “unpleasant flavors”, olfactory “cleanness”, and olfactory “woodiness”. The Eggerhof and Aldino wines had a high rating in terms of “color intensity”, “unpleasant odors”, olfactory “undergrowth”, “overall intensity”, and “warmness”. Also, these wines were highly correlated with the “overall quality judgement”. The Mazzon samples had an olfactory “burning” sensation, “sweetness”, olfactory “spiciness”, and a high “clarity” by visual evaluation. Non-volatile phenols are presented in Figure S3G (Supplementary Materials File S1), and the most intense peaks were identified and are presented in Figure S2 (Supplementary Materials File S1). The variables contributing to the separation of the wines in the F2 factor were z.5 (*m*/*z* 559), z.76 (*m*/*z* 561), z.84 (*m*/*z* 591), z.87 (*m*/*z* 347), z.88 (*m*/*z* 377), and z.101 (*m*/*z* 315) (compounds not yet identified), while those contributing to the separation in the F1 factor were z.51 (*m*/*z* 129), z.68 (*m*/*z* 143), z. 96 (*m*/*z* 183), z.130 (*m*/*z* 173), z.145 (*m*/*z* 366; λmax = 281 nm) and z.147 (*m*/*z* 197; λmax = 277 nm).

#### 2.1.2. Effect of Chitosan Treatment on Mazzon Vineyard

**Figure 2:**
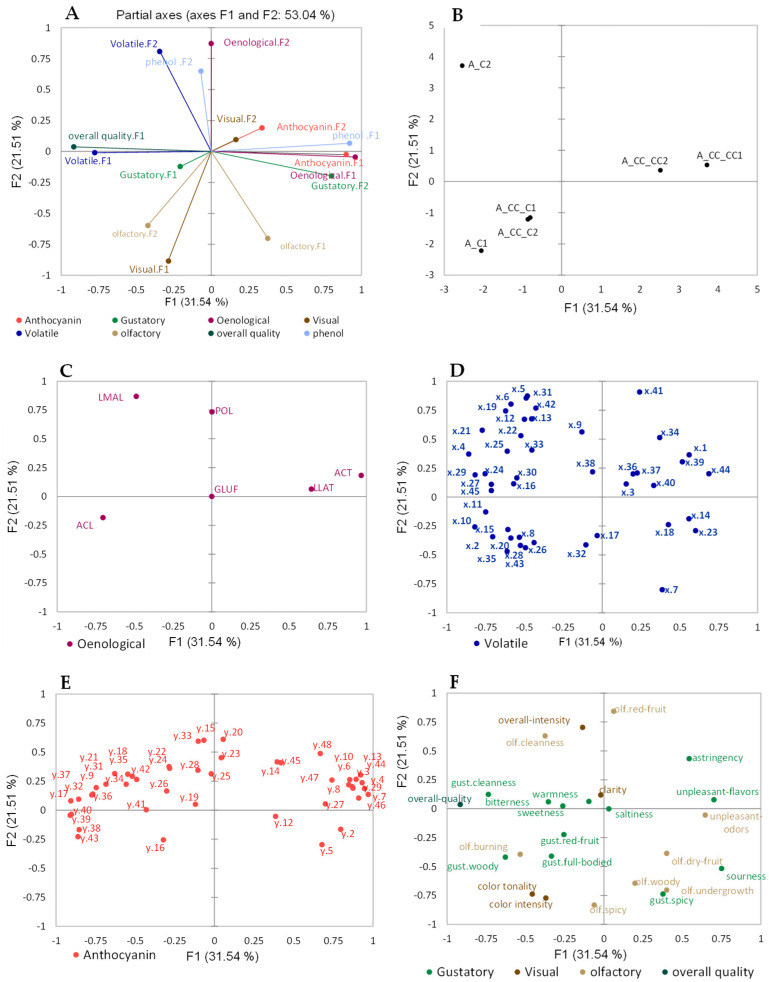
MFA of wines from Mazzon vineyard, evaluating the effect of chitosan treatment on canopy. (**A**) shows the projection on first two MFA partial axes of the Principal Components of the individual datasets, (**B**) score plot, and (**C**–**F**) MFA loading plot showing the separated basic enological variables, volatile compounds, anthocyanins, and sensory analysis variables, respectively. A_C = no treatment with chitosan; A_CC_C = treatment with chitosan only before harvest; A_CC_CC = treatment with chitosan three times a year in the beginning of flowering, end of veraison, and pre-harvest. The identified volatile compounds in (**D**) are presented in Table S1. The most intense peaks of anthocyanins were identified and are presented in Figure S1 (Supplementary Materials File S1). The data are displayed with the replicates (bottle 1 and 2). ACL: acetic acid (g/L); LLAT: lactic acid (g/L); LMAL: malic acid (g/L); ACT: tartaric acid (g/L); GLUF: glucose–fructose (g/L); and POL: total polyphenols (mg/L).

The effect of chitosan treatment on the canopy (Mazzon vineyard) on the resulting quality of Pinot Noir wine was evaluated: Figure 2 shows the MFA of the wines (datasets: basic enological parameters, sensory analysis, volatile compounds, non-volatile phenolics, and anthocyanins). As reported in Figure 2A,B, the first two partial axes accounted for 53.04% of the total variance. The first partial axis, F1, represented 31.54%, while F2 represented 21.51% of the total variability. To carry out a more accurate and clear evaluation, the wine samples were analyzed in replicates. A clear separation in the first partial axis, F1, of the wines from the Mazzon vineyard, which underwent three treatments a year (A_CC_CC), could be observed due to the contribution of phenolic compounds_F1, anthocyanin compounds_F1, basic enological parameters_F1, gustatory attributes_F2, anthocyanin compounds_F2, visual_F2, and olfactory_F1. Similarly, wines from the vineyard treated only once a year (A_CC_C) were distinguished by olfactory F2, gustatory attributes_F1, and visual_F1. Furthermore, wines from the control vineyard (A_C2) were separated due to the volatile compounds_F2 and phenolic compounds_F2.

From the loading plot in Figure 2C, tartaric and lactic acids were highly correlated with the A_CC_CC samples, while acetic acid positively correlated with the A_CC_C samples and was anti-correlated with tartaric and lactic acids. Malic acid contributed to the separation of the control samples. Regarding the volatile profile (Figure 2D), the main compounds contributing to the separation of A_CC_CC were ethyl hexadecanoate (x.44), nerolidol (x.40), furfural (x.14), 2-furanmethanol (x.23), 5-methyl-2-furancarboxaldehyde (x.18), acetaldehyde (x.1), 2(3*H*)-furanone, 5-butyldihydro-4-methyl-, *cis*-whisky lactone (x.39), butanedioic acid, ethyl 3-methylbutyl ester (x.36), *trans*-3-methyl-4-octanolide (*trans*-whisky lactone) (x.34), and n-propyl acetate (x.3). The wine samples A_CC_C could be separated due to the contribution of 3-methyl-1-pentanol (x.8), ethyl acetate (x.2), 1-hexanol (x.11), benzaldehyde (x.15), 1-octanol (x.17), α-terpineol (x.26), anethole (x.32), and α-calacorene (x.35). The most volatile compounds characterizing the A_C2 sample were 3-methyl-1-butanol acetate (x.5), 2-methyl-1-propanol (x.6), 1-propanol (x.4), ethyl 2-hydroxy propanoate (x.9), 2-nonenal (x.12), ethyl octanoate (x.13), butyrolactone (x.21), ethyl decanoate (x.22), ethyl 4-decenoate (x.25), and 2-phenylethyl acetate (x.31).

According to Liu et al. [41], esters such as ethyl hexadecanoate, ethyl octanoate, ethyl decanoate, and ethyl-4-decenoate are described by fruity and floral aromas as well as higher alcohols, such as 1-hexanol, 1-octanol, and 2-methyl propanol, and give a pungent sensation to the wine. A possible explanation for the slightly higher amount of furfural in wines from the vineyard treated with chitosan compared to the control wines (although not significant by ANOVA test) is that furfural and related compounds are known to be produced from the degradation of chitosan itself [42]. It is therefore not to be excluded that the higher abundance of furfural in wines from canopy-treated grapes might be the direct result of a chemical or microbial degradation process of the chitosan during the process of winemaking and/or the aging in oak barriques.

**Figure 3:**
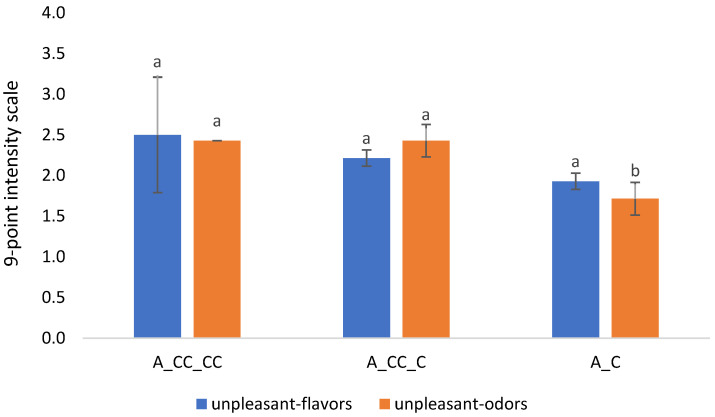
The 9-point intensity score for “unpleasant flavors” and “odors” for wines from Mazzon vineyard evaluating the effect of chitosan treatment on canopy. A_C = no treatment with chitosan; A_CC_C = treatment with chitosan only before harvest; A_CC_CC = treatment with chitosan three times a year in the beginning of flowering, end of veraison, and pre-harvest. Means followed by different letters differ significantly with Tukey’s test (*p* < 0.05). The bar represents the average of three replicates with the respective standard deviation.

Figure 2E shows that A_CC_CC wines are those mostly correlating with the anthocyanin compounds. More particularly, delphinidin-3*O*-glucoside and petunidin-3*O*-glucoside contributed to the separation of these wines, while the ACC_C wines were anti-correlated with most of the anthocyanins. The sensory profile presented in the loading plot in Figure 2F shows that the A_CC_C wines were mainly characterized by gustatory characteristics (“red fruit”, “full body”, “woodiness”), olfactory “burning”, as well as “color tonality” and “color intensity”. Similarly, “unpleasant flavors” and “odors”; “astringency”; “sourness”; olfactory “dry fruit”, “woodiness”, and “undergrowth”; and gustatory “spiciness” were highly correlated with the A_CC_CC wines and contributed to their separation in the first partial axis F1. Gustatory “cleanness”, “bitterness”, and “sweetness” are highly correlated with the “overall quality” and anti-correlated with “unpleasant flavors” and “odors”. In addition, Figure 3 obtained from the ANOVA data highlighted the relation of the chitosan-treated canopies with “unpleasant flavors” and “odors”, and results showed that wines from chitosan-treated canopies and, more particularly, those subjected to multiple treatments per year had a higher rating of “unpleasant flavors” and “odors” compared to the non-treated canopy, which might suggest that grape metabolism is slightly altered compared to untreated grapevines. Figure S4G (Supplementary Materials File S1) shows that wines from the vineyard treated three times a year (A_CC_CC) had high contents of most phenolic compounds, as expected. Several researchers have already demonstrated that the content of polyphenols as well as anthocyanins increased considerably in wines obtained from vineyards treated with chitosan at least three times a year, as detailed in the introduction. Also, similar results were observed with the present study presenting wines from the vineyard treated with chitosan three times a year with a high content of anthocyanins as well as polyphenols. These compounds highly contributed to the separation of the A_CC_CC wines, as seen in Figure 2A. The wine samples A_CC_C from the Mazzon vineyard with only one treatment with chitosan before harvest did not have a high concentration of most phenolic compounds. The same observation was made with wines without treatment, stating that chitosan stimulates the production of polyphenols and anthocyanins in A_CC_CC wines. These results were in line with the work performed by Portu et al. [17] and Duxbury et al. [15], who stated that two treatments with chitosan (one at veraison and the other a week after it) on the canopy barely influenced the phenolic content of the grapes or wine, and a pre-harvest application only of chitosan may not affect the total phenolic and anthocyanin content of grapes and related wine.

### 2.2. Regression Models

#### 2.2.1. Partial Least Squares Regression (PLS-R) for the Overall Quality Judgement (OQJ) of Wines

**Figure 4:**
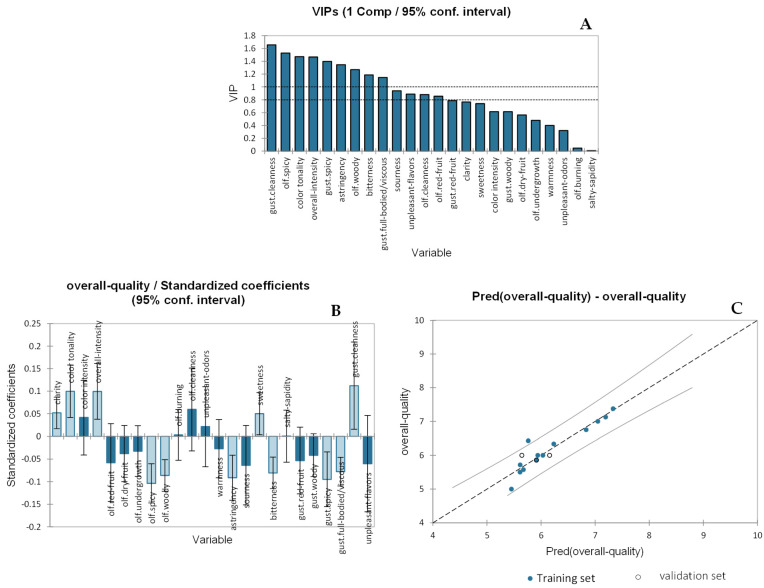
PLS data for the overall quality judgment of wines from four different vineyards considering vineyard location, exposure, and chitosan treatment. (**A**) shows the VIP for the sensory variables, (**B**) shows the effect of the variables on the PLS equation, and (**C**) shows the regression for the OQJ vs. predicted OQJ. In (**A**) the dotted line indicate thresholds (0.8 and 1.0) of the variable importance in projection for variable selection; dashed line in (**C**) indicates the model fitting (predicted vs. experimental), while straight lines indicate the confidence levels (α = 95%) for the same model.

A PLS regression method was applied to study the combination of sensory attributes that have the greatest impact on the overall quality judgement (OQJ) of wines obtained from the four different vineyards as well as those obtained from the Mazzon vineyard with chitosan treatment. Also, post-blend wines (final wine of the winery obtained by mixing wines obtained from treated and non-treated vineyards) were used for model validation. The sensory attributes were treated as the X-matrix (independent explanatory variable), while the y-vector (dependent variable) was OQJ. The model with one component had a Q^2^ (cum) of 0.785, R^2^X (cum) of 0.347, and R^2^Y of 0.881. The VIP graph (variables important in projection) (Figure 4A) allowed us to outline which variables had more influence on the model of the overall quality (arbitrarily with VIP > 1). The most important variables were gustatory “cleanness” (1.66), followed by olfactory “spiciness” (1.53), “color tonality” (1.47), “overall intensity” (1.47), gustatory “spiciness” (1.34), “astringency” (1.35), olfactory “woodiness” (1.27), “bitterness” (1.19), and gustatory “full-body” (1.15). The variables with a positive contribution in the regression model (Figure 4B) were “clarity”, “color tonality”, “overall intensity”, olfactory “burning”, olfactory “cleanness”, “sweetness”, “sapidity”, and gustatory “cleanness”, whereas the variables with a negative effect on the regression were olfactory “red fruit”, olfactory “dry fruit”, olfactory “undergrowth”, olfactory “spiciness”, olfactory “woodiness”, “warmness”, “astringency”, “sourness”, “bitterness”, gustatory “red fruit”, gustatory “woodiness”, gustatory “spiciness”, gustatory “full body”, and “unpleasant flavors”. Finally, Figure 4C represents the experimental observations of the overall quality judgment (OQJ) against the model predictions, showing that one observation is outside the confidence intervals (built at a 95% confidence level). Despite the sample which was outside of the confidence interval, OQJ perfectly modeled the regression with a relatively high R^2^ of 0.881. The full regression model is reported in the Supplementary Materials File S2_OQJ PLS, where 2PN and XPN represent the code of the post-blend sample and are shown in duplicate.

#### 2.2.2. Partial Least Squares Regression to Identify the Volatile Compounds Responsible for the Aroma Sensory Attributes of Wines

**Figure 5:**
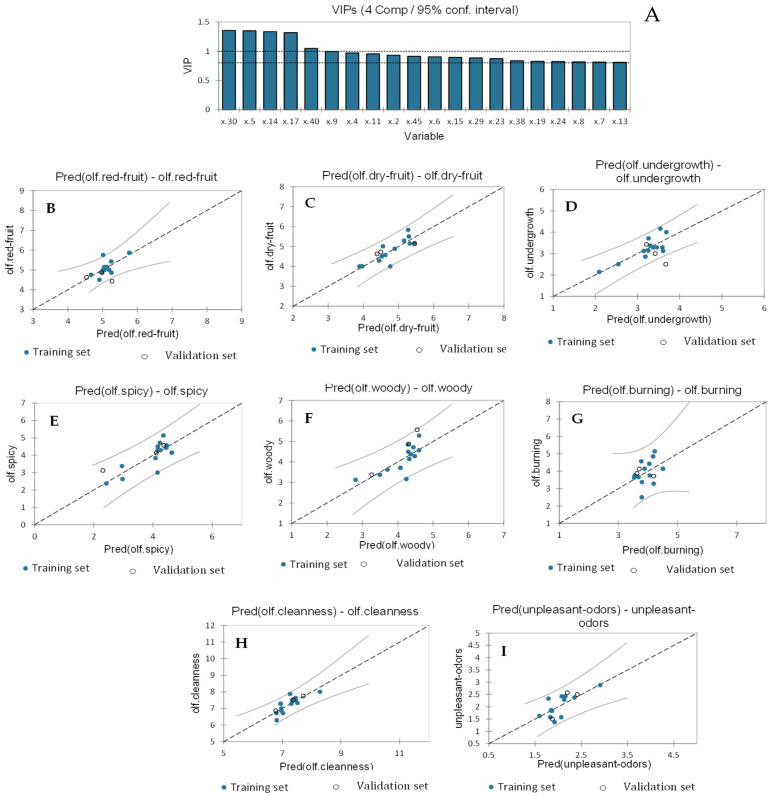
(**A**) shows the variable importance in projection (VIP) in the PLS regression olfactory attributes vs. volatile compounds. (**B**–**I**) show the regression graphs for predicted vs. experimental data for olfactory attributes in wines.

A PLS2 regression model was computed for all the olfactory variables (Y-matrix) versus the volatile variables treated as an X-matrix in order to understand which volatile compounds affected the olfactory sensory attributes taken as a unique profile (not as individual descriptors). The best PLS regression model was calculated for four components and the qualitative index Q^2^ (cum), R^2^X (cum), and R^2^Y (cum); the four components are reported in Table 2. Also, Table 3 reports the R^2^, standard deviation, MSE, and RMSE for each of the olfactory variables used in the regression. The VIP obtained from the PLS2 model are presented in Figure 5A. The most important volatile compounds contributing to the olfactory flavors of the wines are ethyl benzeneacetate (x.30); 3-methyl-1-butanol acetate (x.5); furfural (x.14); 1-octanol (x.17); and nerolidol (x.40). Figure 5B–I show the regression graph for the different olfactory sensory descriptors. The variables with no training set data outside the confidence interval are olfactory “cleanness” (R^2^ of 0.64 and RMSE of 0.28), “unpleasant odors” (R^2^ of 0.52 and RMSE of 0.29), olfactory “burning” (R^2^ of 0.16 and RMSE of 0.62), and olfactory “undergrowth” (R^2^ of 0.67 and RMSE of 0.30). Instead, olfactory “dry fruit” (R^2^ of 0.70 and RMSE of 0.31), olfactory “spiciness” (R^2^ of 0.65 and RMSE of 0.47), olfactory “red fruit” (R^2^ of 0.433 and RMSE of 0.27), and olfactory “woodiness” (R^2^ of 0.56 and RMSE of 0.42) showed 7.69% of the training set samples outside the confidence interval, whereas 33.33% of the validation set samples were found outside the 95% confidence interval for olfactory “undergrowth” and olfactory “red fruit”.

The contribution of volatile profiles on each specific olfactory descriptor is highlighted in Figure S5 (Supporting Information File S1). Volatile compounds with the most positive influence on olfactory “red fruit” are 3-methyl-1-butanol acetate (x.5), ethyl octanoate (x.13), furfural (x.14), 1-octanol (x.17), and ethyl benzeneacetate (x.30), while the negative effect was more given by ethyl acetate (x.2), 1-propanol (x.4), and ethyl-2-hydroxy propanoate (x.9). Olfactory “dry fruit” appears negatively influenced particularly by 3-methyl-1-butanol acetate (x.5), furfural (x.14), 1-octanol (x.17), and ethyl benzeneacetate (x.30), while the positive effect was given by a unique compound, nerolidol (x.40). Regarding olfactory “undergrowth”, the positive contribution was due to ethyl acetate (x.2), 1-propanol (x.4), ethyl 2-hydroxy propanoate (x.9), citronellol (x.29), and nerolidol (x.40), and the negative influence was mostly given by 3-methyl-1-butanol acetate (x.5), followed by ethyl benzeneacetate (x.30), 1-hexanol (x.11), and benzaldehyde (x.15). The positive effect on the olfactory “spiciness” attribute was given by 3-methyl-1-butanol acetate (x.5), ethyl octanoate (x.13), furfural (x.14), 1-octanol (x.17), and ethyl benzeneacetate (x.30), while ethyl 2-hydroxy propanoate (x.9), 3-methyl-1-pentanol (x.8), 3-methyl-1-butanol (x.7), 1-propanol (x.4), and D,L-2,3-butanediol (x.19) mostly contributed negatively. Olfactory “woodiness” was inhibited mostly by 3-methyl-1-butanol acetate (x.5), 1-hexanol (x.11), benzaldehyde (x.15), diethyl butanedioate (x.24), and 2,4-di-ter-butylphenol (x.45), whereas the highest positive effect was given by furfural (x.14) and 1-octanol (x.17). Olfactory “burning” was positively influenced particularly by ethyl benzeneacetate (x.30), followed by 1-octanol (x.17), 3-methyl-1-butanol acetate (x.5), furfural (x.14), and ethyl octanoate (x.13), whereas the main negative contribution was given by nerolidol (x.40). Olfactory “cleanness” was negatively influenced mostly by furfural (x.14), 1-octanol (x.17), and ethyl benzeneacetate (x.30), while a positive effect was mainly given by 1-hexanol (x.11), benzaldehyde (x.15), and 2,4-di-ter-butylphenol (x.45). Finally, “unpleasant odors” was negatively influenced by 3-methyl-1-butanol acetate (x.5), 1-hexanol (x.11), ethyl octanoate (x.13), benzaldehyde (x.15), and 2,4-di-ter-butylphenol (x.45), while many volatile compounds had positive effects, such as furfural (x.14), ethyl 2-hydroxy propanoate (x.9), citronellol (x.29), ethyl benzeneacetate (x.30), 1-octanol (x.17), 1-propanol (x.4), 2-methyl-1-propanol (x.6), and ethyl acetate (x.2).

Sensory attributes that best fitted the regression model were olfactory “dry fruit”, with a relatively high R^2^ of 0.706; this was followed by olfactory “undergrowth”, with an R^2^ of 0.673; olfactory “spiciness”, with an R^2^ of 0.658; and olfactory “cleanness”, with an R^2^ of 0.641. By contrast, those that did not satisfy the model were olfactory “burning”, considered as the worst with a very low R^2^ of 0.163, followed by olfactory “red fruit” (with an R^2^ of 0.433), “unpleasant odors” (with an R^2^ of 0.529), and lastly, olfactory “woodiness” (with an R^2^ of 0.569).

The full regression model is reported in Supplementary Materials File S2: Olfactory PLS.

#### 2.2.3. Partial Least Squares Regression to Identify Non-Volatile Compounds and Enological Parameters Responsible for the Visual and Gustatory Sensory Attributes of Wines

**Figure 6:**
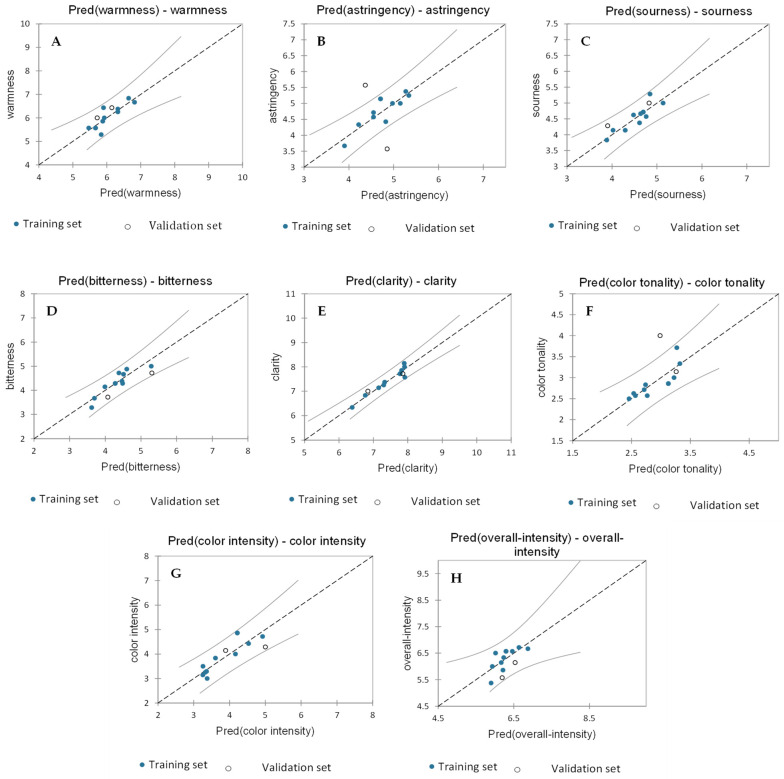
Model-predicted vs. observed visual and gustatory observations for wines. (**A**) “Warmness”, (**B**) ”astringency”, (**C**) ”sourness”, (**D**) ”bitterness”, (**E**) ”clarity”, (**F**) ”color tonality”, (**G**) ”color intensity”, and (**H**) “overall intensity”.

Regarding the visual and gustatory sensory attributes, PLS regression data were used to understand which non-volatile compounds (phenolics and anthocyanins) as well as the enological parameters which had an impact on these sensory attributes. The gustatory sensory attributes were “bitterness”, “warmness”, “astringency”, and “sourness”, whereas the visual attributes were “clarity”, “color tonality”, “color intensity”, and “overall intensity”, which were used as the Y-matrix. The phenolic compounds, anthocyanin compounds, and basic enological parameters (acetic acid, lactic acid, tartaric acid, and total polyphenols) were used as the X-matrix. The best PLS2 regression model was calculated for four components, and the qualitative index of the fourth component had a Q^2^ (cum) of 0.208, R^2^X (cum) of 0.739, and R^2^Y (cum) of 0.761. The “warmness” showed an R^2^ of 0.71 and an RMSE of 0.25, “astringency” had an R^2^ of 0.79 and an RMSE of 0.221, and “sourness” showed an R^2^ of 0.77 and an RMSE of 0.19. A 0.81 R^2^ and 0.21 RMSE were highlighted for “bitterness”, while the visual sensory attribute “clarity” showed an R^2^ of 0.92 and an RMSE of 0.14, “color tonality” had an R^2^ of 0.72 and an RMSE of 0.19, and “color intensity” showed an R^2^ of 0.81 and an RMSE of 0.27. Finally, an R^2^ of 0.52 and an RMSE of 0.28 were registered for “overall intensity” (all the indexes are reported in Table 4). The predicted vs. experimental data for the different visual and gustatory attributes are presented in Figure 6. All the training set and validation set data regarding “warmness”, ”sourness”, ”bitterness”, ”clarity”, ”color intensity”, and “overall intensity” were inside the confidence interval, while “astringency” and “color tonality” showed two and one validation samples outside the confidence interval, respectively. In addition, Figure S6-1 (in Supplementary Materials File S1) shows the variable important in the projection (VIP); the most representative variables were z.32 (Rt. 5.8 min; *m*/*z* 191.0), z.45 (Rt. 7.4 min; *m*/*z* 181.0), and acetic acid. Figure S6 from 2–33 shows the standardized coefficient effect in the regression.

Most of these sensory attributes perfectly modeled the regression with a relatively high R^2^, particularly the “clarity” attribute, with an R^2^ of 0.925, followed by “color intensity” (R^2^ of 0.818), “bitterness” (R^2^ of 0.814), “astringency” (R^2^ of 0.797), “sourness” (R^2^ of 0.778), “color tonality” (R^2^ of 0.721), and “warmness” (R^2^ of 0.719). The only attribute that did not fit the model well was “overall intensity”, with a low R^2^ of 0.520.

The full regression model is reported in Supplementary Materials File S2: Visual and gustatory PLS.

### 2.3. Analysis of Variance (ANOVA)

#### 2.3.1. Effect of the Vineyards

A one-way ANOVA test considering the effects of the vineyards was applied to the general enological parameters, the volatile compounds, non-volatile phenols, anthocyanins, and the sensory profiles to provide a more in-depth explanation of the interplay of variables using the MFA. For brevity, only the variables showing significant differences are here reported (α = 0.05). Table S2 (Supplementary Materials File S1) shows the results of the one-way ANOVA test from the general enological parameters, considering the vineyard as a variable. The following parameters were analyzed: acetic acid (g/L); lactic acid (g/L); malic acid (g/L); tartaric acid (g/L); sum of glucose–fructose (g/L); and total polyphenols (mg/L). The enological parameters were in line with the expected values [43,44,45]. The amount of acetic acid averaged between 0.23 and 0.34 g/L. While malic acid underwent malolactic fermentation (average of 0.08 g/L), significant differences were observed for lactic and tartaric acids, where tartaric acid (2.04 g/L) was high in Patone (D) samples compared to the other three vineyards. On the other hand, lactic acid was found low in Mazzon (A) and Patone (D) wines and differed from Eggerhof (E) wines, which had a high concentration (2.67 g/L), followed by Aldino (2.17 g/L).

One-way ANOVA was performed to highlight significant anthocyanins present in wines from the four vineyards, and the results are reported in Table S3 (in Supplementary Materials File S1). Considering the compounds at retention times of 12.33 min (y.24) and 14.06 min (y.33), the variable “altitude” significantly affected these compounds, with a high concentration in wines obtained from vineyards located at high altitudes (800 and 1050–1150 m.a.s.l.) compared with the wines from the Mazzon (A) vineyard (only 350 m.a.s.l.). More particularly, the effect of “altitude” was highlighted with malvidin-3O-glucoside (y.14; *m*/*z* 493, 331), showing a clear distinction from high to low altitude. The Eggerhof (E) vineyard, located at the maximum altitude (1050–1150 m.a.s.l.), had a considerably high amount, followed by the Aldino (C) and Patone (D) (800 m.a.s.l.) samples and lastly the Mazzon (A) samples. Considering the anthocyanin vitisin A (y.16; *m*/*z* 561, 399), the Aldino and Eggerhof vineyards had a higher quantity than the other vineyards. These two vineyards are exposed to the south, so the ANOVA suggests that the variable “exposure” may affect (e.g., increase) the quantity of vitisin A in wine. Regarding peonidin-3O-glucoside (y.13; *m*/*z* 463) and an unidentified anthocyanin at 12.74 min (y.26; *m*/*z* 525), the variables “altitude” and “exposure” were not significant.

On the other hand, the anthocyanin compounds (not yet identified) at retention times of 11.98 min (y.22); 12.23 min (y.23); 13.06 min (y.27); 13.22 min (y.28); 13.59 min (y.31); 14.54 min (y.36); 14.70 min (y.37); 14.82 min (y.38); 14.98 min (y.39); and 15.82 min (y.43) showed a unique characteristic in the way they affected the four wines. High concentrations were observed in the Aldino samples, followed by the Patone and Eggerhof samples, in which both vineyards were associated with the same concentration for each compound, but these three vineyards did not show any statistically significant difference among them. Instead, the significant difference was highlighted between the Mazzon and Aldino samples, showing the Mazzon vineyardʹs wines with a relatively low amount. The opposite effect was observed with the compound at 16.29 min (y.45). Considering these compounds, the variable “altitude” could be influential. Taking into account all the significant variables, wines from the Mazzon vineyard showed a considerably low amount of anthocyanins compared to other vineyards, highlighting that low altitude can minimize the concentration of these compounds in wines due to the shorter exposure to the sunlight of the valley floors.

The one-way ANOVA performed on the sensory descriptors to highlight the significant differences among wines obtained from the four vineyards is reported in Table S4 (in Supplementary Materials File S1). For better readability, only the attributes with a significant difference with Tukey’s test *p* < 0.05 are reported. The sensory attributes with significant differences were “clarity”, defined as the absence of veiling or suspension in the wine, and the Mazzon vineyard’s (A) wines had the higher “clarity” score, an average of 7.79 out of the 9-point intensity score, which significantly differed from the Eggerhof (E) vineyard, which had the lowest score (an average of 6.58). By contrast, a reverse effect was observed with olfactory attributes such as “dried fruits” and “undergrowth” as well as “warmness” (a warm sensation perceived in the mouth due to the effect of alcohol), with a higher intensity score for the Eggerhof vineyard and the lowest value for the Mazzon vineyard.

Table S5 (Supplementary Materials File S1) shows the one-way ANOVA for the phenols. The most intense and significant phenols identified that differentiated the wine samples were *p*-coumaroylquinic acid (z.47), glutathionyl caftaric acid (z.114), coutaric acid (z.133), and astilbin (z.151), with a high concentration of astilbin and *p*-coumaroylquinic acid in Patone wines, while low concentrations were observed in Eggerhof wines for both compounds. Similarly, Aldino wines showed a high content of glutathionyl caftaric acid and coutaric acid, with the lowest concentration in Mazzon wines regarding glutathionyl caftaric acid.

#### 2.3.2. Effect of Chitosan Treatment on Mazzon Vineyard

A series of one-way ANOVAs was performed on the basic enological parameters, the volatile compounds, phenols, anthocyanins, and sensory profiles, considering the effect of chitosan treatment on wines from the Mazzon vineyard. To simplify the results, most tables represent just the variables with a significant difference (α = 0.05).

The one-way ANOVA performed on the basic enological parameters considering the effect of chitosan treatment on the Mazzon vineyard is presented in Table S6 (in Supplementary Materials File S1). The significant variables were tartaric and acetic acids. The amount of tartaric acid in wines from the Mazzon vineyard treated with chitosan three times a year (beginning of flowering, end of veraison, and pre-harvest: A_CC_CC) was significantly higher than in wines obtained in the control vineyard (no treatment: A_C) or treated just once a year (before harvest: A_CC_C). On the contrary, acetic acid was found high in Mazzon wines treated once with chitosan, followed by the wines from the control. The wines obtained with treated grapes over all the year had a significantly lower amount than those obtained in the vineyard treated just once. As acetic acid is produced during or after fermentation during winemaking, the concentration found in different wines may not be related just to the vineyard location. The volatile compounds listed in Table S1 were used to evaluate the effect of chitosan treatment on wines obtained from the Mazzon vineyard. A one-way ANOVA was applied considering chitosan treatment on the canopy as factor. Results highlighted two volatile compounds with a significant difference with Tukey’s test: (*p* < 0.05) citronellol (x.29) and 2,4-di-tert-butylphenol (x.45). Citronellol and 2,4-di-tert-butylphenol showed to be higher in the wines that were not submitted to any treatment (A_C), approximatively double compared to the treated samples. Particularly, no statistical difference could be observed between both treatments regarding 2,4-di-tert-butylphenol, but differences were observed in the control wines with a significantly high concentration. Instead, control wines and the wines from the canopy treated just once had similarly high concentrations of citronellol and differed from wines treated three times a year and showed a relatively low concentration.

Table S7 (Supplementary Materials File S1) represents the one-way ANOVA test of significance with Tukey’s test (*p* < 0.05) for anthocyanins present in wines from the Mazzon vineyard. All the significant compounds for instant delphinidin-3O-glucoside (y.4), petunidin-3O-glucoside (y.10), and peonidin-3O-glucoside (y.13), apart from y.38 (14.82 min) and y.43 (15.82 min), showed a considerably high concentration in the wines obtained from canopy treated three times a year and differed significantly from those without any treatment or treated just once. Instead, no statistical difference was observed between untreated wines and those treated just once a year. On the contrary, anthocyanins at retention times of 14.82 min (y.38) and 15.82 min (y.43) were found low in the wines treated several times and differed significantly from the others, whereas no statistical difference was observed between wines from untreated canopy or with treatment carried out only one time a year.

The ANOVA test performed on the sensory data showed that three attributes significantly contributed to the separation of the wines, namely olfactory “woodiness”, gustatory “woodiness”, and “unpleasant odors”. Wines from the canopy treated with chitosan only one time and three times a year had a relatively high intensity score (an average of 5 out of 9) for olfactory “woodiness” perception compared to the control wines (an average score of 4). Instead, the gustatory “woodiness” was more perceived in wines treated just one time followed by the control wines. On the other hand, wines from both treatments (canopy treated one and three times a year) had a relatively high intensity score for “unpleasant odors” (an average of 2.4 out of the 9-point intensity score) and differed from the control wines, which had a 1.7 score.

The one-way ANOVA performed on the phenolic compounds is presented in Table S8 (Supplementary Materials S1). The two most intense phenols identified that differentiated the wines were *trans*-caftaric acid (z.117) and catechin (z.134), which were high in Mazzon wines treated three times a year and differed from the control wines. The samples that underwent just one treatment a year were not significantly different from the control. By contrast, almost all the most intense phenols identified, such as P-coumaroylquinic acid (z.47), gallic acid (z.89), glutathionyl caftaric acid (z.114), procyanidin dimer-1 (z.127), coutaric acid (z.133), procyanidin dimer-2 (z.136), epicatechin (z.142), *trans*-piceid (z.148), and astilbin (z.151) did not present any significant differences among the wines. Instead, most of the significant phenols were of lower intensity and not yet identified, such as z.9 (*m*/*z* 310.9 at 4.3 min), z.28 (*m*/*z* 133.0 at 5.6 min), z.42 (*m*/*z* 605.1 at 6.5 min), z.84 (*m*/*z* 591.0 at 15.3 min), z.93 (*m*/*z* 342.9 at 17.5 min), z.102 (*m*/*z* 593.0 at 21.7 min), z.104 (*m*/*z* 456.9 at 21.8 min), z.108 (*m*/*z* 379.0 at 23.2 min), z.117 (*m*/*z* 310.9 at 24.6 min), z.126 (*m*/*z* 129.0 at 28.2 min), z.134 (*m*/*z* 577.0 at 30.7 min), and z.149 (*m*/*z* 478.9 at 43.6 min), showing a high concentration in wines obtained from the vineyards with chitosan treatment, particularly those from the vineyards which underwent three treatments a year, whereas no significant difference could be observed between the wines from untreated vineyards and those from the treatment only one time a year. This result correlated with the research performed by Tessarin et al. [11], who showed that canopy treated three times (at the beginning, end of veraison, and pre-harvest) with chitosan caused a significantly high quantity of non-volatile compounds in Cabernet Sauvignon wines compared to the same wine from the untreated vineyards. The increase in phenols can be the response of the plant as chitosan is considered to mime the presence of pests. Also, the present study was in line with the work performed by Duxbury et al. [15], highlighting the fact that only a pre-harvest application of chitosan in the vineyard may not affect the total phenolic and anthocyanin content of the grapes and related wine.

By contrast, an adverse effect was observed in the phenolic compounds z.29 (*m*/*z* 199.0 at 5.6 min) and z.40 (*m*/*z* 173.0 at 6.3 min), with a relatively low amount in wines from the vineyards with several treatments, which differed significantly from the other two wines. The following significant compounds, z.13 (*m*/*z* 310.9 at 4.6 min), z.54 (*m*/*z* 237.0 at 8.6 min), z.69 (*m*/*z* 151.0 at 11.8 min), z.88 (*m*/*z* 376.9 at 16.2 min), z.118 (*m*/*z* 494.9 at 25.4 min), and z.125 (*m*/*z* 175.0 at 28.2 min), did not adhere totally to the research conducted by Duxbury et al. [15], showing that wines from the pre-harvest application of chitosan may also have a considerably high concentration of these compounds as well as those obtained from the vineyard with several treatments, which differed from the control wines (no treatment), which had a low amount.

## 3. Materials and Methods

### 3.1. Vineyards and Treatments on Canopy

Wines destined for commerce were obtained from four different vineyards of the Franz Haas SrL winery (Montagna, Bozen-Bolzano, Italy). The vineyards are located in different areas of the Trentino Alto-Adige region (Italy). They are in Mazzon (Ora), Patone (near Rovereto, Trento), Aldino (a small village in the province of Bolzano), and Eggerhof (close to the village of Aldino). Additionally, for one specific vineyard (Mazzon), the effect of the treatments with chitosan on the canopy was evaluated. The vinification processes for each mass were performed separately for each vineyard. Two bottles of wine were collected from each vineyard (2 bottles × 4 vineyards = 8 bottles), and for the chitosan treatment, 2 bottles × 2 treatments = 4 bottles were collected, and the 2 bottles from an untreated plot of the vineyard were used as control. These wines were stored for six months in bottles at room temperature before the analysis. The wine obtained from the four vineyards without chitosan treatment, also called control wines, were A (Mazzon); C (Aldino); D (Patone); and E (Eggerhof). The samples with chitosan treatments on the canopy were A_CC_C (Mazzon vineyard with only one treatment with chitosan before the harvest) and A_CC_CC (Mazzon with chitosan treatment three times: at the beginning of flowering, end of veraison, and pre-harvest). After the vinification of each mass, this winery proceeded with the blending of the different samples to create their final assembled wines, which are normally blends of wines from different vineyards, treated and non-treated. This post-blend sample was included in the regression model of the present study, and the reference codes of the biological replicate were 2PN and XPN. The list of different wines analyzed, along with the related codes as well as specific information on the vineyards, is summarized in Table 5.

### 3.2. Winemaking

The grapes were destemmed without pressing on arrival in the cellar. The fermentation took place in stainless steel tanks, all around 90 hL in volume, that were filled up to 80% of the volume and were kept at controlled temperature around 24 °C. During the fermentation (with maceration), pumping-over and plunging were carried out daily. After 10 days, the mass was racked. Then the decantation started, and after some days of stabilization, the wine was separated from the lees. The wines were then transferred and stored in barriques (Mazzocchi, 2020) [4]. As reference for these winemaking procedures, the samples are A (Mazzon vineyard); C (Aldino); D (Patone); and E (Eggerhof).

Concentrated pectolytic enzymes (Ever Srl, via Pacinotti 37, Pramaggiore, Italy) were added to all samples to increase color extraction. Gall tannins and chestnut tannins (2 g/hL; ExperTi, via Colomba 14) were added to all the samples except for the chitosan thesis (A_CC_C and A_CC_CC), in which glicotan and oak tannin (2 g/hL; Bioenologia 2.0 Srl, via Verdi 32, Oderzo, Italy) were instead added; these additions were performed during fermentation at the top of the vessel. SO_2_ was not added to the grapes, but 7 g/hL was added at the end of the malolactic fermentation, just before the transfer to barriques. Then, after one month and a half, 3 g/hL of sulfites was added to the barriques [4].

### 3.3. Analytical and Statistical Methods

#### 3.3.1. General Enological Parameters

A multiparametric wine analyzer, MIURA One (Exacta&Optech, San Prospero, Modena, Italy), was used to evaluate the basic enological parameters of the wines. Before the analysis, a calibration curve against reference standards was recorded. All samples were analyzed after filtration with 0.22 µm syringe filters. Wine samples were analyzed for the following parameters: acetic acid; lactic acid; malic acid; tartaric acid; sum of glucose and fructose; total polyphenol content; and free and total SO_2_.

#### 3.3.2. HPLC Analysis of Non-Volatile Profile

The phenolic profile of the wines was characterized by HPLC-DAD/FLD, as described by [46,47]. Briefly, an ODS column (Eurosphere II, C18 stationary phase, 250 × 4.6 mm × 5 µm, Knauer, LabService Analytica, Anzola dell’Emilia, Bologna, Italy) installed on a Nexera X2 UHPLC system (Shimadzu, Milano, Italy) equipped with a UV-Vis diode array detector (DAD, sampling rate 12.5 Hz, time constant = 0.320 s, scan range = 200–800 nm, 1.2 nm slit width) and fluorescence detector (FLD, 10 Hz sampling rate, λexc = 276 nm, λem = 316 nm, with 1× gain) in series was used. The HPLC mobile phase was formed by solvent A (0.1% formic acid in degassed milliQ water) and solvent B (0.1% formic acid in acetonitrile). The gradient method was 0–2.5 min 1% B, 2.5–50 min 1–25% B, 50–51 min 25–99% B, 51–55 min 99% B, 55–56 min 99–1% B, 56–60 min 1% B. The HPLC flow rate was 0.7 mL/min. The HPLC peaks were reported as integrated areas vs. retention times using the automatic integration provided by the software (LabSolutions, Shimadzu). The peak alignment was performed manually. Tentative compound identification was conducted by full-scan mass spectrometry determination and the relative PDA λmax assignment to classify the compounds at least in a phenolic class where a complete identification could not be achieved. Solutions of standard compounds (gallic acid, *trans*-caffeic acid, p-coumaric acid, *trans*-caftaric acid, (+)-catechin, (−)-epicatechin, *trans*-piceid, protocatechuic acid, astilbin, coutaric acid, glutathionyl caftaric acid, and procyanidin B2) were analyzed by standard injection, and their PDA spectra, MS/MS spectra, and retention times (min) were used as references. The tentative identification of phenolic compounds is shown in Figure S2.

The anthocyanin profile was analyzed on a UHPLC-QqQ/MS instrument (Agilent LC/TQ 6465 system, Milan, Italy). The instrument was equipped with a 1260 Infinity II UHPLC with a quaternary pump system and a 1260 Infinity II WR PDA detector in series to an AJS ESI QqQ mass analyzer (Agilent). The separations were conducted on a Poroshell 120, SB-C18 2.1 mm × 100 mm × 2.7 µm (Agilent Technologies Italia, Milan, Italy) kept at 30 °C at a 0.35 mL/min flow rate. Mobile phases: (A) 0.1% formic acid in ultrapure water and (B) 0.1% formic acid in MS-grade acetonitrile. The separations were run in gradient mode. Gradient program: 0–2.5 min 1% B, 2.5–50 min 1–25% B, 50–51 min 25–99% B, 51–55 min 99% B, 55–56 min 99–1% B, 56–60 min 1% B. The injection volume was 5 µL. The ESI source was run in positive mode, applying the following instrumental parameters: gas temperature 260 °C, gas flow 4 L/min, nebulizer pressure 35 psi, sheath gas heater 300 °C, sheath gas flow 12 L/min, capillary voltage +2500 volt (V). For the anthocyanins in Pinot Noir, the corresponding MS traces were identified by looking for the precursor ions and for specific in-source fragments, e.g., *m*/*z* 493 and *m*/*z* 331 for malvidin-3-*O*-glucoside and its aglycone, respectively. Figure S1 shows the target identification of anthocyanins in Pinot Noir based on molecular ion and lambda max (UV spectrum absorbance).

#### 3.3.3. HS-SPME-GCxGC-ToF/MS

For the gas chromatographic analysis of the volatile components, the samples were prepared following an already published specific protocol [48]. In detail, for each sample, 0.5 g of sodium chloride was added in a 10 mL GC vial sealed with a perforable screw-cap, and 4 mL of wine was transferred into the vial with the NaCl. Before the analysis, 10 µL of internal standard (IS) was added. The IS used was 2-methyl-3-pentanol (pre-diluted 1:50 in ethanol). The sample was then pre-incubated at 40 °C for 30 min. Analytes in the wine headspace were then adsorbed for 15 min onto a 1 cm SPME fiber coated with a ternary stationary phase (DVB/CAR/PDMS; 50/30 µm). Finally, the analytes were desorbed for 6 min in the GC injector heated at 240 °C. For the GCxGC bidimensional analysis, the instrument was a GCxGC Agilent 7890B coupled with ToF-MS Pegasus^®^ Flux BT 4D (LECO Corporation, Berlin, Germany). The column in the first dimension was a PEG phase MEGA-WAX-Spirit (MEGA S.r.l, Italy) 40 m × 180 µm × 0.30 µm. For the second dimension, the column was an Rxi^®^-17Sil phase (Restek Corporation, Lisses, France) 1.1 m × 100 µm × 0.10 µm. Helium was used as the carrier gas. The separation was performed in spitless mode at constant flow with 1 mL/min flow rate. The septum purge flow was set at 2 mL/min, whereas the inlet purge was programmed at 6 mL/min for 6 min. The temperature of the inlet was set at 240 °C. Temperature program: 40 °C for 6 min, then 3 °C/min to 180 °C. Then, the rate was increased to 10 °C/min to reach 240 °C. The second oven was maintained at the main oven temperature +5 °C. The modulation period was 2.5 s, with an injection time of 0.08 s. The transfer line temperature and ion source temperature both were 250 °C, the rate of acquisition of spectra was 150 spectra^.^ s^−1^, and mass range of the ToF was set to *m*/*z* 35–530, with an analyzer frequency of 32 kHZ. The SPME sample preparation, sample injection, sample acquisition, and data processing was managed in ChromaToF^®^ software (ver.2021, LECO Corporation, Germany). The dataset was then processed (features alignment and compounds assignment) using ChromaTOF^®^-Tile, applying a tentative compound identification method using the library from the NIST database (NIST 2017, version 2.3). Retention indexes were calculated against the series of C4–C22 ethyl esters of linear saturated fatty acids (ethyl acetate to ethyl arachidate).

#### 3.3.4. Sensory Analysis

A panel of 13 subjects (7 females and 6 males, ages 23 to 35) took part in a 10 h training for the sensory analysis at the Free University of Bozen-Bolzano. An informed consent was signed by the participants and no monetary reward was given. No ethics procedure was conducted as the study was low risk because the samples were commercially produced and the panelists were instructed to spit the samples so the quantity of alcohol for each session was under the tolerance threshold (25.87 mL). The panelists received detailed instructions from the panel leader on the definition of the sensory descriptors and how to conduct the sensory evaluation before each session. The sensory descriptors were generated during the qualitative phase by the panel. The final sensory lexicon used to evaluate the samples was formed by visual, olfactory, and gustatory descriptors, as shown in Table 6. The samples were evaluated according to the Quantitative Descriptive Analysis (QDA^®^, Tragon Corporation, Arlington, TX, USA) procedure, as described in UNI 10957:2003 using a nine-point intensity scale. The panel was also asked to give an overall quality judgment (OQJ), defined as the objective score from 0 to 9 points about the quality of the wines. For each session, six bottles of wine stored at room temperature were opened just before the analysis and were offered randomly to the panelists in ISO glasses (30 mL/glass) codified with three-digit numbers in two different sessions (one biological replicate/session). Crackers with low salt were provided as a palate cleanser.

#### 3.3.5. Statistical Analysis

Statistical analysis was performed using XLStat (version 2023.2.1414, Addinsoft, Paris, France), with statistical significance determined using an alpha value of 0.05 unless otherwise stated. The datasets for the two factors evaluated (vineyard location and chitosan treatment on canopy) were basic enological parameters, sensory analysis (visual, olfactory, gustatory, overall quality judgment), volatile compounds, non-volatile phenolic compounds, and anthocyanins. Multiple Factor Analysis (MFA) was applied to explore the main trends differentiating the samples and the experimental variables with a set of eight continuous variables: wine basic enological parameters, volatile compounds, phenolic compounds, anthocyanins, and wine sensory profile divided by the different modes of perception (color, gustatory, olfactory, and overall quality judgment). Also, regression models were built on the dataset to highlight which variable contributed most to characterizing the wines, and lastly, one-way ANOVA (analysis of variance) followed by Tukey’s HSD (honest significant difference) for post hoc mean separation were used to identify the sensory and chemical variables which were significantly influenced by the study factors.

## 4. Conclusions

This research evaluated the effect of vineyard location, exposure, and agronomic management (chitosan treatment on canopy) on the volatile, non-volatile, and sensory profiles as well as the general enological parameters of South Tyrolean Pinot Noir wines. The samples were produced from four different vineyards in the Trentino-Alto Adige region (Mazzon, Aldino, Patone, and Eggerhof). The wines from the four vineyards were analyzed and compared focusing on their location and exposure, while those obtained from a single vineyard (Mazzon) were studied to evaluate the effect of chitosan treatment on canopy and were compared with a control sample (no treatment).

Considering the wines obtained from the four vineyards without chitosan treatment (control) and evaluating the effect of vineyard location and exposure, the results of the enological parameters show that lactic and tartaric acids were significantly different (*p* < 0.05) among wines with a high concentration of tartaric acid in Patone, while a high concentration of lactic acid was found in Eggerhof wines, followed by Aldino wines, with a relatively low amount in Mazzon and Patone wines. The volatile compounds presented a high concentration of the most significant variables in wines from Mazzon. Also, this trend was observed in the PCA plot, where Mazzon wines showed a high correlation with most identified compounds. Similar observations were highlighted for non-volatile phenols, where out of the four most significant variables, considering a *p*-value < 0.0001, three compounds, z.3 (*m*/*z* 195.0 at 4.1 min), z.4 (*m*/*z* 390.9 at 4.1 min), and z.10 (*m*/*z* 191.0 at 4.3 min), had a relatively high concentration in Mazzon wines, indicating that low altitude (350 m.a.s.l.) could favor the production of these compounds in wine. On the contrary, wines from the Mazzon vineyard showed a considerably low intensity of the most significant anthocyanins compared to the other vineyards located at high altitudes (800 and 1050–1150 m.a.s.l.), emphasizing that low altitude can minimize the concentration of these compounds in wines. The effect of vineyard location was also observed in the sensory evaluation, where significant differences were observed between the highest altitude wines (Eggerhof: 1050–1150 m.a.s.l) and the lowest ones (Mazzon: 350 m.a.s.l.), with a high perception of olfactory “dry fruit”, olfactory “undergrowth”, and “warmness” in wines from high altitudes. By contrast, wines from low altitudes had a high intensity score for “clarity” descriptors. Multiple Factor Analysis showed a clear separation of Mazzon and Patone wines from the F1 and F2 axes, respectively, whereas Aldino and Eggerhof were grouped. The most significant aroma compounds contributing to the separation of Mazzon wines were higher alcohols, esters, and terpenes, whereas ketones, esters, and alcohols distinguished Aldino and Eggerhof, and finally, Patone wines were separated mostly by esters.

Regarding the effect of chitosan treatment on the Mazzon vineyard, the basic enological parameters showed a significant difference among wines regarding tartaric and acetic acids. A high amount of tartaric acid was highlighted in wines that underwent several treatments a year and differed from the others. Instead, acetic acid was found high in wines treated once a year, followed by the control wine, whereas a low concentration was found in wines treated several times. Two aroma compounds were statistically significant, showing control wines with a considerably high amount of citronellol and 2,4-di-tert-butylphenol, approximatively double compared to the treated samples. The PCA graph showed a high correlation of control wines (no treatment) with most of the identified volatile compounds, and they were separated from treated wines along PC1. By contrast, considering non-volatile components such as anthocyanins, almost all the significant variables were found higher in wines obtained from the canopy treated three times a year. Non-volatile phenols were higher in wines from treated canopy, showing that chitosan stimulated the production of these compounds in wine. A similar trend was also observed by Iriti et al. [12], where the total polyphenol and antioxidant activity of grapes and wines obtained from vineyards with chitosan treatment was higher compared to untreated controls and fungicide-treated samples. This increase in phenols can be the response of the plant as chitosan is considered to mime the presence of pests in the vineyard. Sensory evaluation presented three significant descriptors, and a relatively high-intensity score was given for olfactory “woodiness” descriptors in treated samples differing from the control wines, whereas gustatory “woodiness” was more perceived in wines treated just one time, followed by the control wines. The results of the MFA also showed wines from the Mazzon vineyard treated three times a year had the greatest correlation with most non-volatile phenols and were completely separated from the others.

Regression models were built to understand the variables that most contributed to characterizing the wines. A PLS1 regression model was applied to the sensory descriptors to evaluate the most impactful ones on the overall quality judgment and results and showed that gustatory “cleanness”, olfactory “cleanness”, “overall intensity”, “color tonality”, “color intensity”, “sweetness” and “clarity” contributed positively to the overall quality judgment, whereas olfactory (“spiciness”, “woodiness”, “red fruit”), “astringency”, “bitterness”, gustatory “spiciness”, “full body”, and “unpleasant flavors” affected them negatively. Olfactory sensory descriptors are associated with the volatile compounds, whereas gustatory and visual descriptors are more affected by the non-volatile compounds and enological parameters. In this regard, PLS2 regression models were built to identify the volatile compounds responsible for the flavor sensory attributes of wines; the most important volatile compounds contributing to the olfactory flavors of the wines were ethyl benzeneacetate, 3-methyl-1-butanol acetate, furfural, and 1-octanol, while the most important variables in the projection contributing to visual and gustatory descriptors were z.32 (Rt. 5.8 min; *m*/*z* 191.0), z.45 (Rt. 7.4 min; *m*/*z* 181.0), and acetic acid.

The present study has helped in highlighting the fact that canopy treated with chitosan at least three times a year could increase considerably the quantity of non-volatile compounds in wines. Future research could be conducted to evaluate the evolution of these non-volatile compounds over time.

## Figures and Tables

**Table 1:** One-way ANOVA for significant differences with Tukey’s test (*p* < 0.05) for volatile compounds in wine samples from Mazzon vineyard (A), Aldino vineyard (C), Patone vineyard (D), and Eggerhof vineyard (E). Numbers in bold represent the volatile compounds as listed in Table S1. a, ab, b, and c letters near the numbers indicate the samples grouping obtained by the Tukey’s test.

Wines	x.2	x.3	x.4	x.8	x.9	x.10	x.14	x.15	x.17	x.18	x.20	x.21
A	6,675,747 ab	8,283,127 ab	335,017 b	540,694 a	4,093,904 b	3,644,990 b	14,585,331 a	1,387,724 b	3,873,619 a	2,434,583 a	630,189 a	3,446,505 a
D	4,404,294 b	6,303,866 b	478,534 b	442,361 ab	6,755,748 a	9,455,400 a	14,190,181 a	1,102,369 b	1,453,939 b	1,991,437 a	249,516 ab	1,767,459 b
C	8,159,445 a	11,139,104 a	432,010 b	328,940 ab	4,504,241 ab	9,524,711 a	1,585,906 c	639,003 b	2,300,074 ab	502,922 b	199,338 b	1,437,020 b
E	6,713,101 ab	10,129,613 a	875,120 a	147,125 b	6,785,969 a	11,889,788 a	8,042,353 b	2,687,260 a	1,597,135 b	1,729,145 a	111,249 b	1,524,666 b
Pr > F	0.032	0.010	0.002	0.031	0.025	0.007	0.001	0.007	0.032	0.011	0.023	0.020
**Wines**	**x.25**	**x.27**	**x.29**	**x.30**	**x.33**	**x.36**	**x.37**	**x.38**	**x.39**	**x.41**	**x.45**	
A	206,836 ab	533,270 a	54,788 a	399,051 a	3,842,883 b	23,396,506 a	11,231,772 a	584,371,828 a	655,435 ab	846,206 b	125,279 a	
D	585,677 a	334,621 b	26,328 ab	196,123 b	5,780,058 a	17,854,483 a	8,683,904 a	443,420,094 ab	557,048 b	239,291 b	82,145 b	
C	195,029 b	385,117 ab	41,174 ab	253,891 b	6,329,581 a	17,126,232 ab	8,344,102 a	417,364,280 b	610,509 ab	3,849,583 a	91,588 ab	
E	183,237 b	224,939 b	11,073 b	156,599 b	4,108,870 b	10,000,704 b	4,803,874 b	229,152,184 c	956,862 a	435,358 b	94,559 ab	
Pr > F	0.032	0.008	0.045	0.006	0.003	0.010	0.005	0.004	0.049	0.000	0.039	

**Table 2:** Quality index (Q^2^, R^2^Y, and R^2^X) for partial least squares regression on olfactory sensory descriptors.

Statistic	Comp. 1	Comp. 2	Comp. 3	Comp. 4
Q^2^ cum	0.078	0.133	0.098	0.120
R^2^Y cum	0.195	0.344	0.447	0.546
R^2^X cum	0.531	0.687	0.834	0.916

**Table 3:** Quality index (R^2^, standard deviation, MSE, RMSE) for PLS regression on olfactory sensory descriptors.

Statistic	Olfactory Red Fruit	Olfactory Dry Fruit	Olfactory Undergrowth	Olfactory Spiciness	Olfactory Woodiness	Olfactory Burning	Olfactory Cleanness	Unpleasant Odors
R^2^	0.433	0.706	0.673	0.658	0.569	0.163	0.641	0.529
Std. deviation	0.352	0.403	0.385	0.609	0.547	0.797	0.366	0.377
MSE	0.076	0.100	0.091	0.228	0.184	0.391	0.083	0.088
RMSE	0.276	0.316	0.302	0.478	0.429	0.626	0.287	0.296

**Table 4:** Quality index (R^2^, standard deviation, MSE, and RMSE) of PLS regression model on the wines’ visual and gustatory data.

Statistic	Warmness	Astringency	Sourness	Bitterness	Clarity	Color Tonality	Color Intensity	Overall Intensity
R^2^	0.719	0.797	0.778	0.814	0.925	0.721	0.818	0.520
Std. deviation	0.364	0.312	0.272	0.309	0.205	0.274	0.387	0.398
MSE	0.066	0.049	0.037	0.048	0.021	0.038	0.075	0.079
RMSE	0.257	0.221	0.192	0.219	0.145	0.194	0.274	0.282

**Table 5:** Vineyard codes, altitudes, exposure, and applied treatment. A = Mazzon vineyard; C = Aldino vineyard; D = Patone vineyard; E = Eggerhof vineyard. These codes, A, C, D, and E, represent the vineyards with no chitosan treatments; A_CC_C = treatment with chitosan only before harvest; A_CC_CC = treatment with chitosan all year.

Vineyard	Vineyard Code	Vineyard Altitude (m.a.s.l.)	Vineyard Exposure	Treatment on the Canopy
Mazzon	A	350	Northwest	None
Mazzon	A_CC_C	350	Northwest	Chitosan once a year: before harvest
Mazzon	A_CC_CC	350	Northwest	Chitosan three time a year: beginning of flowering, end of veraison, and pre-harvest
Aldino	C	800	South	None
Patone	D	800	East	None
Eggerhof	E	1050–1150	South	None
Mazzon, Aldino, Patone, Eggerhof	Wine code 2PN and XPN	/	/	Blend of treated and non-treated wines

**Table 6:** Sensory descriptors used to evaluate wine samples with their respective definition.

Sensory Descriptors	Descriptors	Definition
VISUAL		
Clarity		Absence of particles in suspension
Color tonality	Red garnet or orange/brown	Tonality or shade of color
Color intensity	Red garnet or orange/brown	Intensity of color
OLFACTORY		
Red fruit	Strawberry, blackberry, raspberry, blackcurrant	Fruit with red or black skin from forest
Dried fruits	Strawberry jam, raisin, prune, fig	Jam from strawberry or another red fruit; dry raisin, prune, or fig
Undergrowth	Mushroom, wet wood, musk, fern	From forest undergrowth, a complex of different odors from mushroom, wet wood in the forest, or musk
Spiciness	Clove, black pepper, anise and liquorice	Different types of spices; clove (strong balsamic odor), black pepper (pungent odor), anise, and liquorice (balsamic odor)
Woodiness	Vanilla, oak, and coffee	Vanilla phenolic odor (sweeter, reminiscent of cake), oak (resinous odor), coffee (burnt odor)
GUSTATORY		
Warmness	Alcohol	Warm sensation perceived in the mouth due to alcohol
Astringency/ tannicity	Tannins or alum	Puckering mouthfeel caused by the tannins, precipitation of saliva, and dry in the mouth
Sourness	Acid: citric, lactic, tartaric	Having an acid taste resembling that of vinegar, lemon juice, etc.
Sweetness	Sucrose, glucose, fructose	Effect typically from sugar
Bitterness	Caffeine	Taste sensation that is peculiarly acrid, like coffee
Saltness/sapidity	Salt or glutamate	Has a salty taste
Red fruit	Strawberry, blackberry, raspberry, blackcurrant, cherry	Having a strawberry flavor or another flavor from red fruit
Woodiness	Vanilla, oak, and coffee	Vanilla phenolic odor (sweeter, reminiscent of cake), oak (resinous odor), coffee (burnt odor)
Full-bodied/viscous	High concentration of sugar, oil, or fat	Visual characteristic and chemestasis characteristics; full mouthfeel or sensation from fatty foods

## Data Availability

The data presented in this study are available on request from the corresponding author.

## Supplementary Materials

The following supporting information can be downloaded at: https://www.mdpi.com/article/10.3390/molecules29091916/s1, Supporting Information File S1: Table S1 represents the volatile compounds identified in wines and listed according to their elution order. LRI = linear retention index; Figure S1: Target identification of anthocyanins in Pinot Noir based on molecular ion [M-H]—(*m*/*z*) and lambda max (UV spectrum absorbance); Figure S2: Tentative compound identification by off-line LC-DAD-QqQ-MS with their respective code used in the present study; Figure S3 represents the MFA of wines from the four different vineyards. (A) shows the projection of the Principal Components of the different datasets applied on the first two MFA partial axes, (B) represents the observation plot, and (G) shows non-volatile phenolic compounds. Identified phenolic compounds are presented in Figure S2. In Figure S3-B, the symbol (A) means Mazzon vineyard, (C) Aldino vineyard, (D) Patone vineyard, and (E) Eggerhof vineyard; Figure S4 shows the MFA of wines from Mazzon vineyard evaluating the effect of chitosan treatment on canopy. (A) shows the projection on first two MFA partial axes of the Principal Components of the individual datasets, (B) score plot, and (G) shows non-volatile phenolic compounds. Identified phenolic compounds are presented in Figure S2 (Supplementary Materials File S1). A_C = no treatment with chitosan; A_CC_C = treatment with chitosan only before harvest; A_CC_CC = treatment with chitosan three times a year in the beginning of flowering, end of veraison, and pre-harvest. The most intense peaks of phenolics were identified and are presented in Figure S2; Figure S5: PLS-R for the volatile compounds with the olfactory sensory data; Figure S6: PLS-R for the non-volatile phenolic and anthocyanin compounds, basic enological parameters with the visual and gustatory data; Table S2: One-way ANOVA of the enological parameters. Numbers in bold mean significant differences with Tukey’s test (*p* < 0.05). A: Mazzon vineyard; C: Aldino vineyard; D: Patone vineyard; and E: Eggerhof vineyard; Table S3 represents the one-way ANOVA of significant differences with Tukey’s test (*p* < 0.05) for anthocyanins in wine samples. Mazzon vineyard (A), Aldino vineyard (C), Patone vineyard (D), and Eggerhof vineyard (E). Only the significant compounds are presented and those which could not be identified are represented with their retention time in min (numbers in bold) and their respective code; Table S4: One-way ANOVA for the sensory descriptors. Only the attribute with a significant difference with Tukey’s test (*p* < 0.05) are presented. Mazzon vineyard (A), Aldino vineyard (C), Patone vineyard (D), and Eggerhof vineyard (E); Table S5 represents the one-way ANOVA for only the significant phenolics in wine with Tukey’s test (*p* < 0.05). A: Mazzon vineyard; C: Aldino vineyard; D: Patone vineyard; and E: Eggerhof vineyard. The most intense and significant phenols identified are p-coumaroylquinic acid (z.47); glutathionyl caftaric acid (z.114); coutaric acid (z.133); and astilbin (z.151), reported in bold. Meanwhile, low-intensity phenols not yet identified are reported using codes (z) and are not in bold; Table S6: One way-ANOVA of the enological parameters. Numbers in bold mean significant difference with Tukey’s test (*p* < 0.05). A_C = no treatment with chitosan; A_CC_C = treatment with chitosan only before harvest; A_CC_CC = treatment with chitosan all year; Table S7 represents the one-way ANOVA for significant differences with Tukey’s test (*p* < 0.05) for anthocyanins in wines from Mazzon vineyard. A_C = no treatment with chitosan; A_CC_C = treatment with chitosan only before harvest; A_CC_CC = treatment with chitosan all year. Only the significant compounds are presented, and those which could not be identified are represented with their retention time (Rt) in min and their respective code; Table S8 represents the one-way ANOVA with Tukey’s test (*p* < 0.05) for only the significant phenolic compounds in wine from Mazzon vineyard. A_C = no treatment with chitosan; A_CC_C = treatment with chitosan only before harvest; A_CC_CC = treatment with chitosan all year. The most intense and significant phenols identified are *trans*-caftaric acid (z.117) and catechin (z.134), reported in bold. Meanwhile, low-intensity phenols not yet identified are reported using codes (z) and are not in bold; Supporting Information File S2: QQJ PLS; Olfactory PLS; Visual and Gustatory PLS.
