# Supplementary material for: Terroir Dynamics: Impact of Vineyard and Canopy Treatment with Chitosan on Anthocyanins, Phenolics, and Volatile and Sensory Profiles of Pinot Noir Wines from South Tyrol
Source: Molecules. 2024 Apr 23;29(9):1916. doi: 10.3390/molecules29091916 (PMC11085818; doi:10.3390/molecules29091916)
Supplement: Supplementary file 1 [file molecules-29-01916-s001.zip › Supporting Information _File S1.pdf]

SUPPORTING INFORMATION

Table S1 represents the volatile compounds identified in wines and listed according to their elution order.
LRI = Linear retention index

Code	Volatile compounds	LRI (Ref./NIST)	Base peak (m/z)	fragmentation pattern (m/z)
x.1	Acetaldehyde	692 [21]	29	29; 43; 44
x.2	Ethyl Acetate	891 [21]	43	43; 45; 61; 70
x.3	n-propyl acetate	969 [22]	43	43; 61; 73
x.4	1-propanol	1040 [23]	31	31; 42; 59
x.5	1-butanol, 3-methyl, acetate	1124 [24]	43	43; 55; 70; 87
x.6	1-propanol, 2-methyl-	1092 [24]	43	39; 41; 42; 43; 74
x.7	1-butanol, 3-methyl-	1209 [24]	55	41; 42; 43; 55; 70
x.8	1-pentanol, 3-methyl-	1331 [21]	56	41; 43; 55; 56; 69
x.9	Propanoic acid, 2-hydroxy-, ethyl ester	1341 [25]	45	29; 45; 75
x.10	2H-pyran-2-one, tetrahydro-3,6-dimethyl-	/	/	/
x.11	1-hexanol	1356 [24]	56	41; 42; 43; 55; 56; 69
x.12	2-nonenal	1537 [26]	41; 43	41; 43; 55; 70; 83
x.13	Octanoic acid, ethyl ester	1444 [21]	88	75; 60; 73; 88; 101; 127
x.14	Furfural	1466 [24]	96	38; 39; 95; 96
x.15	Benzaldehyde	1527 [24]	77	51; 77; 105; 106
x.16	2(1H)-naphthalenone,3,4,4a,5,6,7-hexahydro-1,1,4a-trimethyl-	/	/	/
x.17	1-octanol	1564 [24]	56	41; 43; 55; 56; 69; 70; 84
x.18	2-furancarboxaldehyde, 5-methyl-	1597 [21]	53	53; 109; 110
x.19	D,L-2,3-butanediol	1620 [27]	45	41; 43; 44; 45; 55; 57
x.20	Bicyclo[3.1.0]hexan-2-ol, 2-methyl-5-(1-methylethyl)-, (1α,2α,5α)-	1459 [22]	93	43; 71; 91; 93; 121; 136
x.21	Butyrolactone	1640 [28]	42	41; 42; 56; 86
x.22	Decanoic acid, ethyl ester	1648 [21]	88	73; 88; 101
x.23	2-furanmethanol	1665 [28]	98	41; 53; 81; 98
x.24	Butanedioic acid, diethyl ester	1687 [21]	101	55; 73; 101; 129
x.25	4-decenoic acid, ethyl ester, (Z)-	/	29	29; 41; 55; 69; 88; 110
x.26	α-terpineol	1695 [24]	59	59; 93; 121; 136
x.27	1-Propanol, 3-(methylthio)-	1708 [22]	106	49; 57; 58; 61; 106
x.28	Naphthalene,1,2,3,5,6,8a-hexahydro-4,7-dimethyl-1-(1-methylethyl)-, (1S-cis)-	1758 [29]	161	105; 119; 134; 161
x.29	Citronellol	1754 [30]	69	41; 55; 67; 69; 81; 95
x.30	Benzeneacetic acid, ethyl ester	1776 [31]	91	39; 65; 91; 164
x.31	Acetic acid, 2-phenylethyl ester	1822 [24]	104	43; 91; 104
x.32	Anethole	1834 [32]	148	77; 105; 117; 147; 148
x.33	Benzyl alcohol	1885 [24]	79	51; 77; 79; 107; 108
x.34	trans-3-methyl-4-octanolide (trans-whisky lactone)	/	99	41; 42; 43; 69; 71; 99

x.35	α-calacorene	1911 [33]	/	/
x.36	Butanedioic acid, ethyl 3-methylbutyl ester	1892 [34]	/	/
x.37	1,6-heptadien-3-yne	/	91	63; 65; 91; 92
x.38	Phenylethyl alcohol	1919 [24]	91	65; 91; 92; 122
x.39	2(3H)-Furanone, 5-butyldihydro-4-methyl-, cis-whisky lactone	/	/	/
x.40	Nerolidol	1992 [35]	69	41; 43; 69; 71; 93; 107
x.41	Phenol, 4-ethyl-	2210 [21]	107	77; 107; 122
x.42	Naphthalene, 1,6-dimethyl-4-(1-methylethyl)-	2242 [36]	183	153; 168; 183; 198
x.43	Methyl anthranilate	2257 [37]	119	65; 92; 119; 120; 151
x.44	Hexadecanoic acid, ethyl ester	2288 [21]	88	41; 43; 55; 57; 88; 101
x.45	2,4-di-tert-butylphenol	2321 [36]	191	57; 191; 206

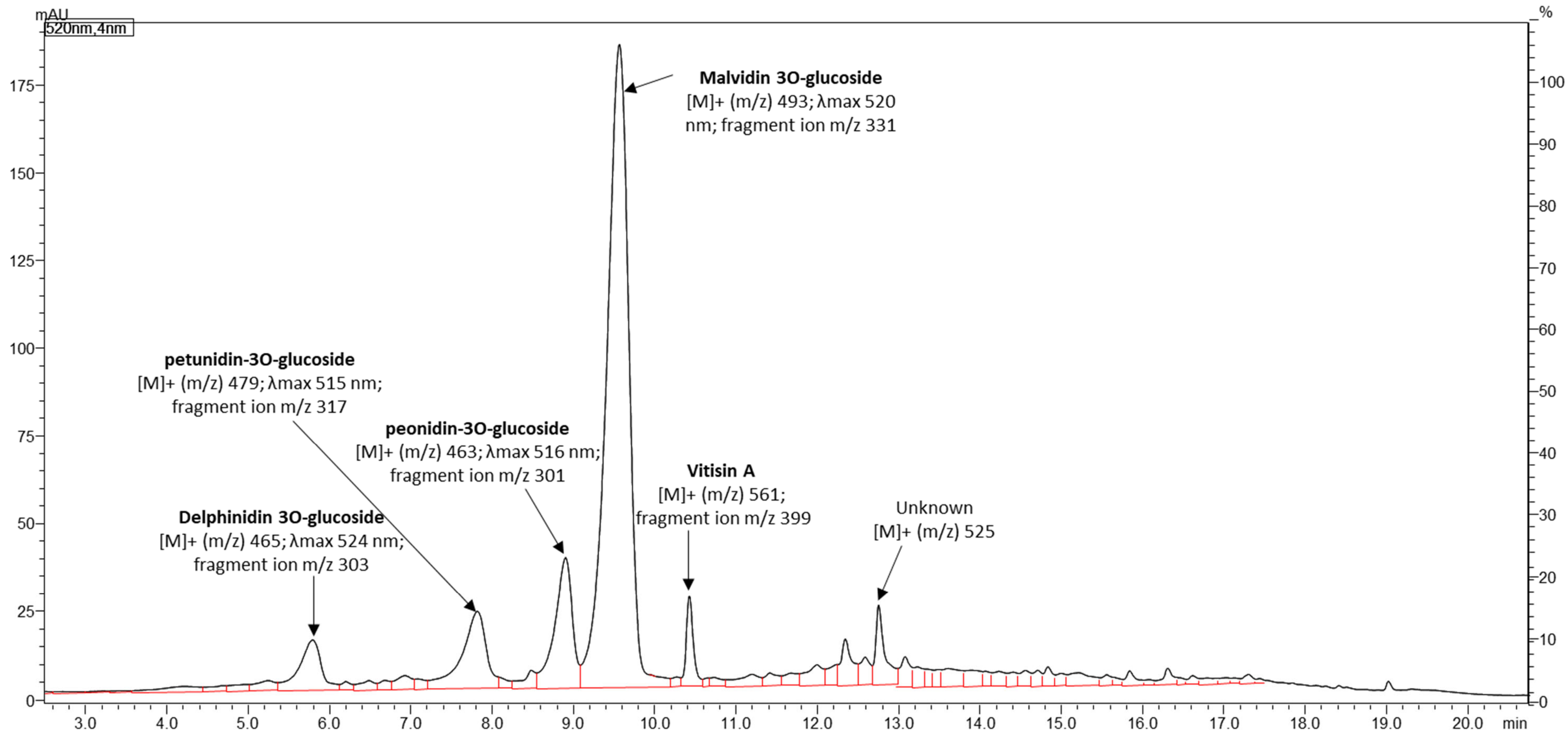

Figure S1: Target identification of anthocyanins in Pinot Noir based on molecular ion [M-H] – (m/z) and lambda max (UV spectrum absorbance)

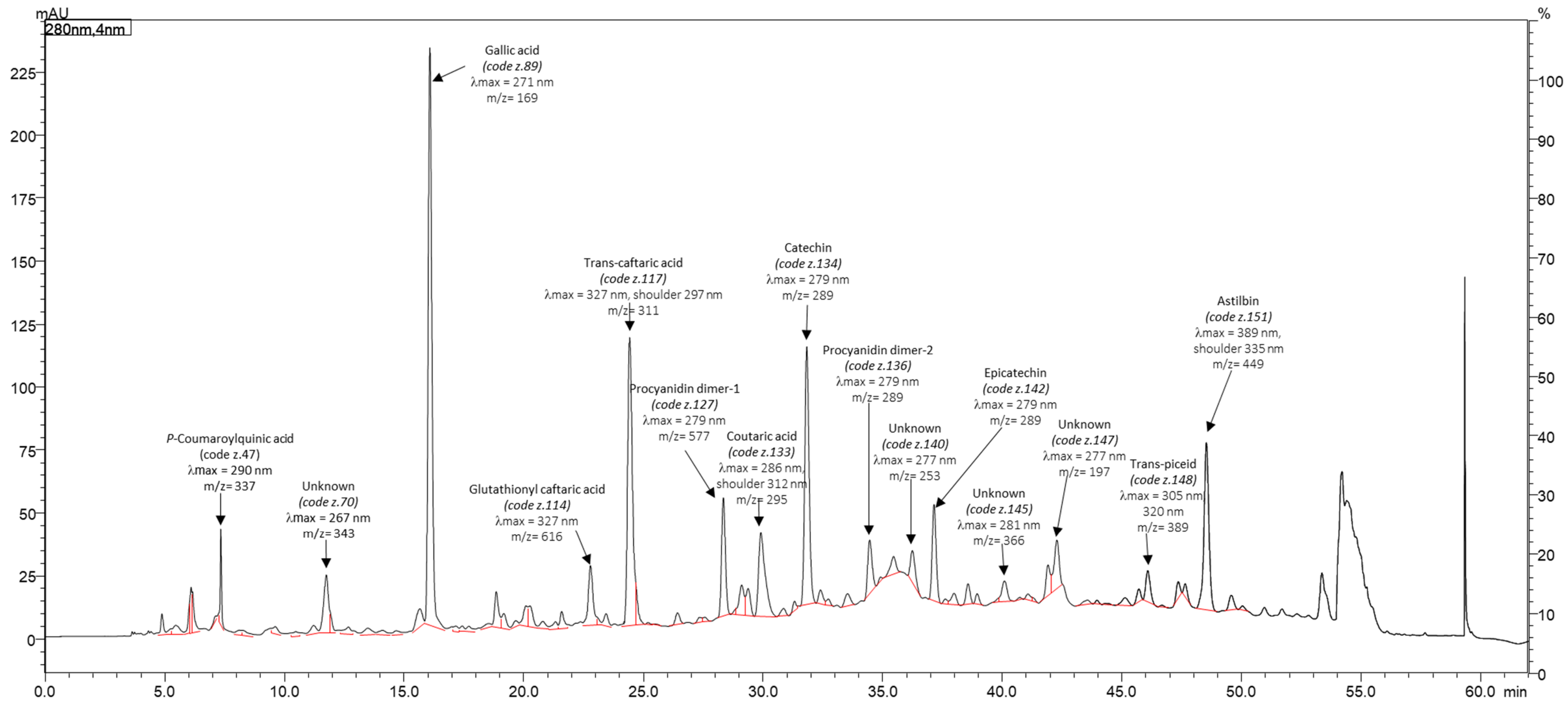

Figure S2: Tentative compounds identification by off-line LC-DAD-QqQ-MS with their respective code used in the present study.

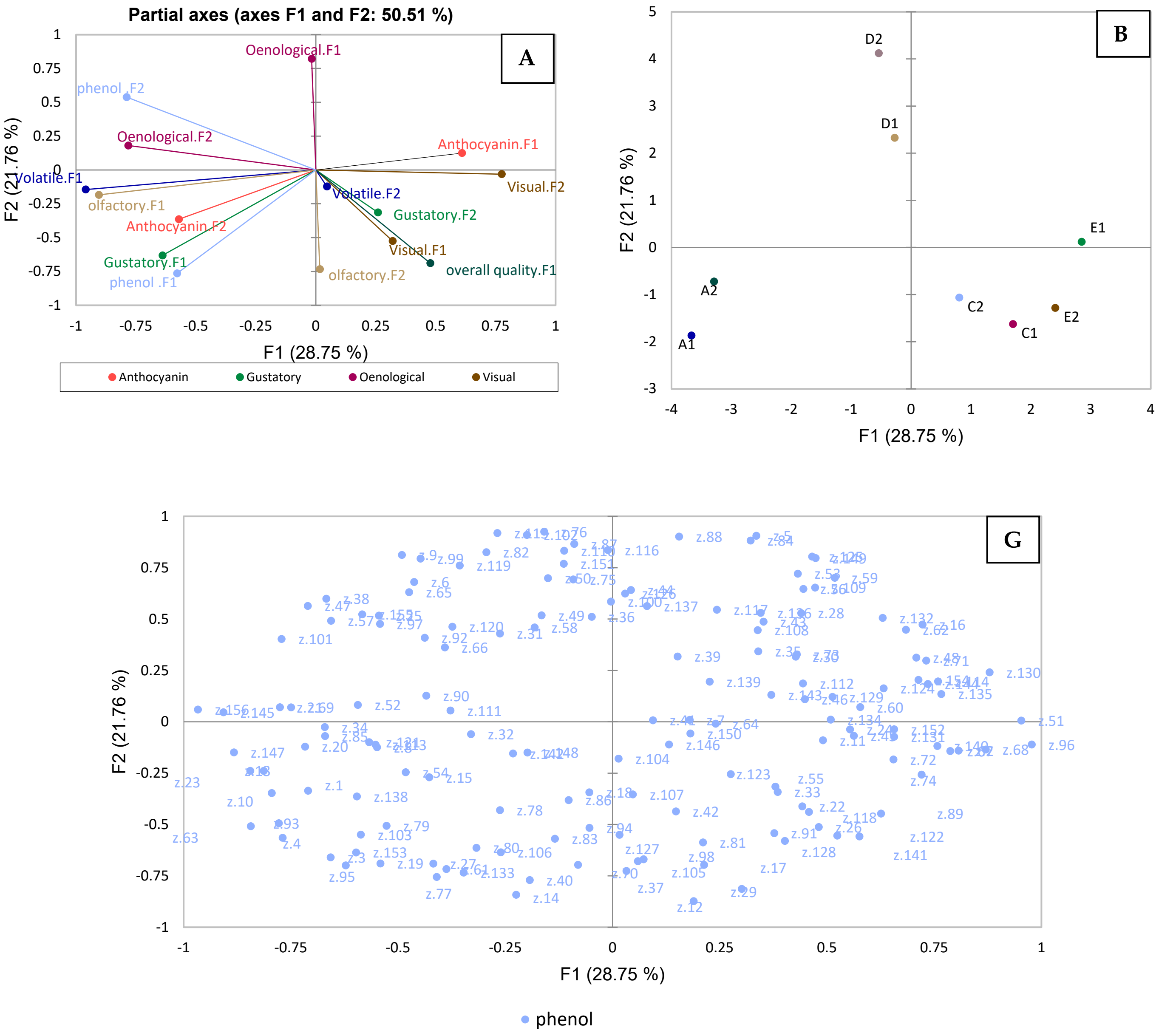

Figure S3 represents the MFA of wines from the four different vineyards. (A) shows the projection of the Principal Components of the different datasets applied on the first two MFA partial axes, (B) represents the observation plot, and (G) shows non-volatile phenolic compounds. Identified phenolic compounds are presented in Figure S2. In Figure S3-B the symbol (A) means Mazzon vineyard, (C) Aldino vineyard, (D) Patone vineyard, and (E) Eggerhof vineyard.

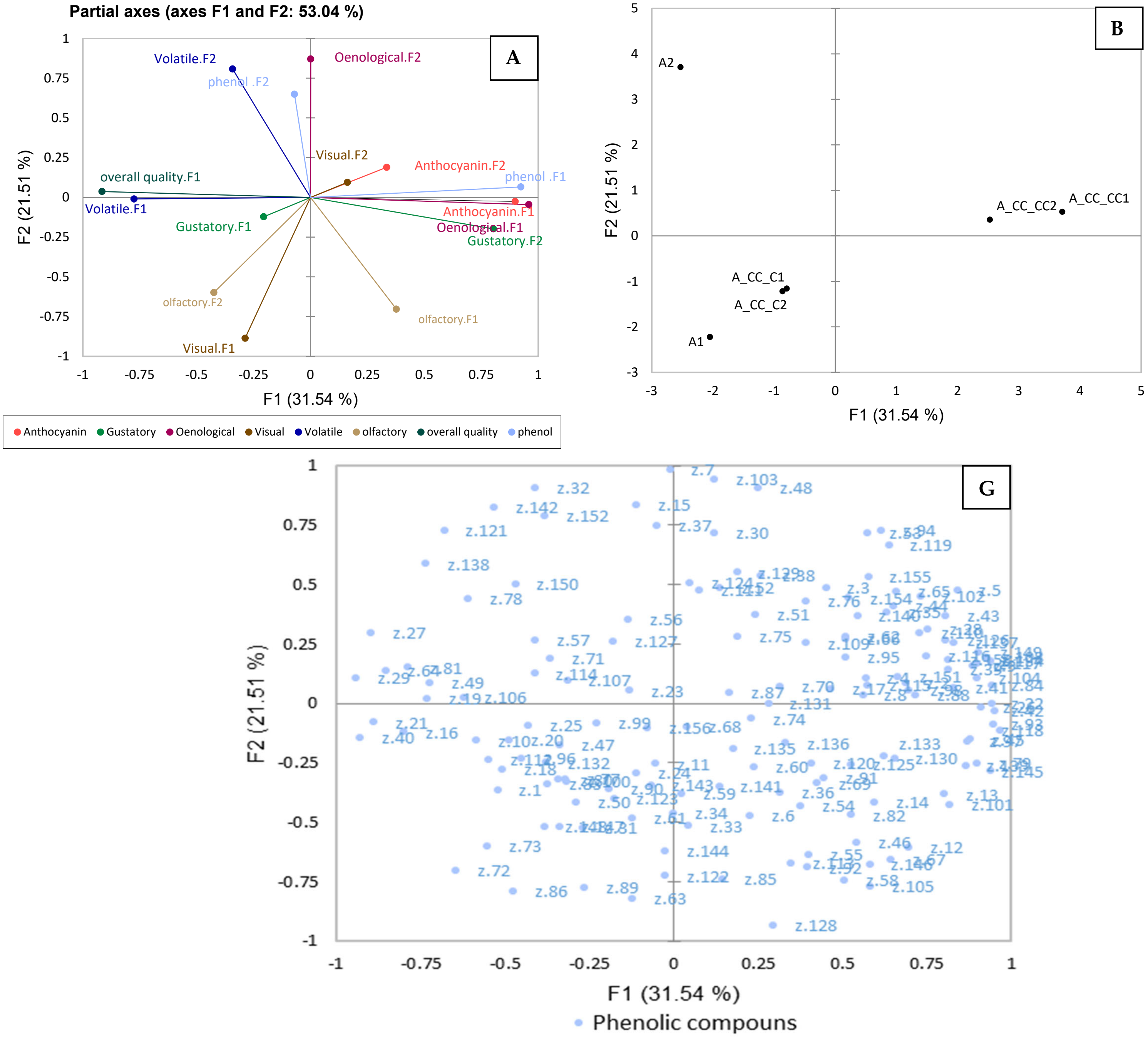

Figure S4 shows the MFA of wines from Mazzon vineyard evaluating the effect of chitosan treatment on canopy. (A) shows the projection on first two MFA partial axes of the Principal Components of the individual datasets, (B) Score plot, and (G) shows non-volatile phenolic compounds. Identified phenolic compounds are presented in Figure S2 (Supporting Information File S1). A_C = no treatment with chitosan; A_CC_C = treatment with chitosan only before harvest; A_CC_CC = treatment with chitosan three times a year: beginning of flowering, end of veraison and pre-harvest. The most intense peaks of phenolic were identified and are presented in Figure S2.

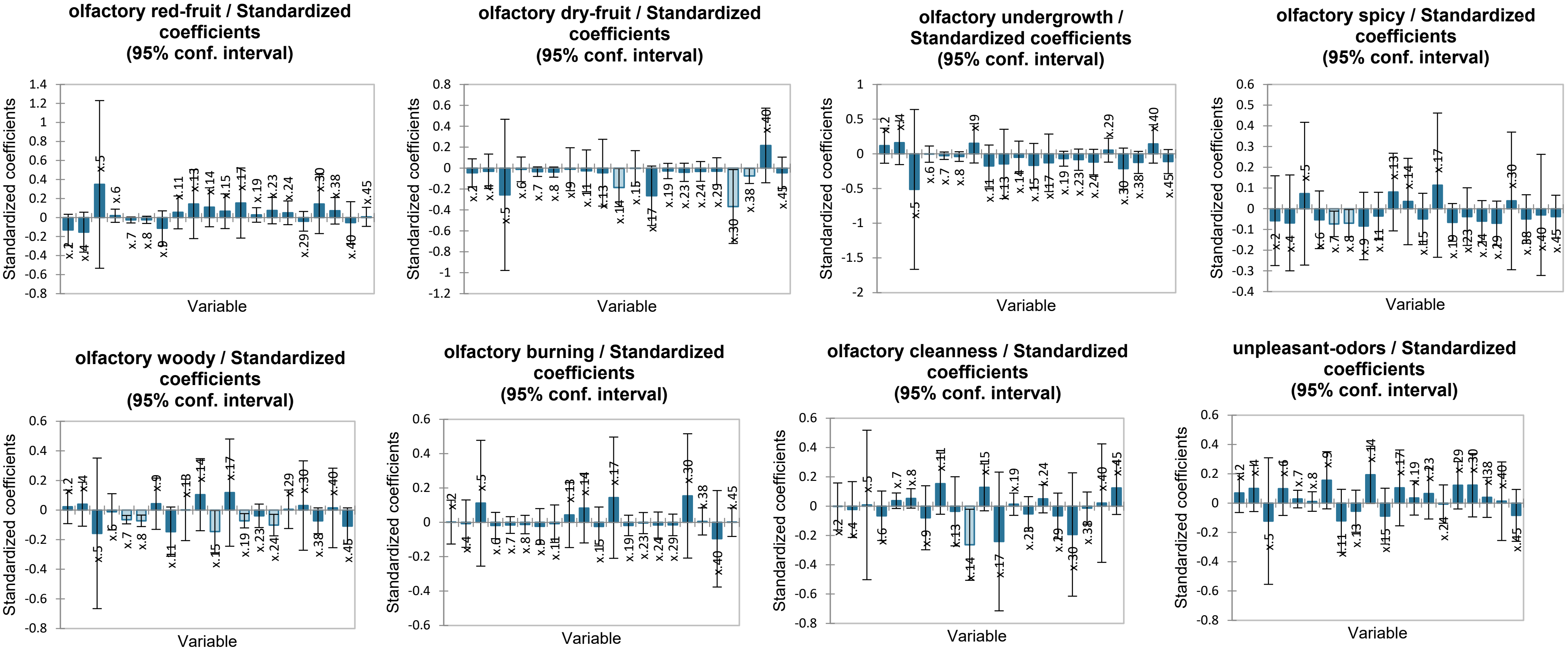

Figure S5. PLS-R for the volatile compounds with the olfactory sensory data

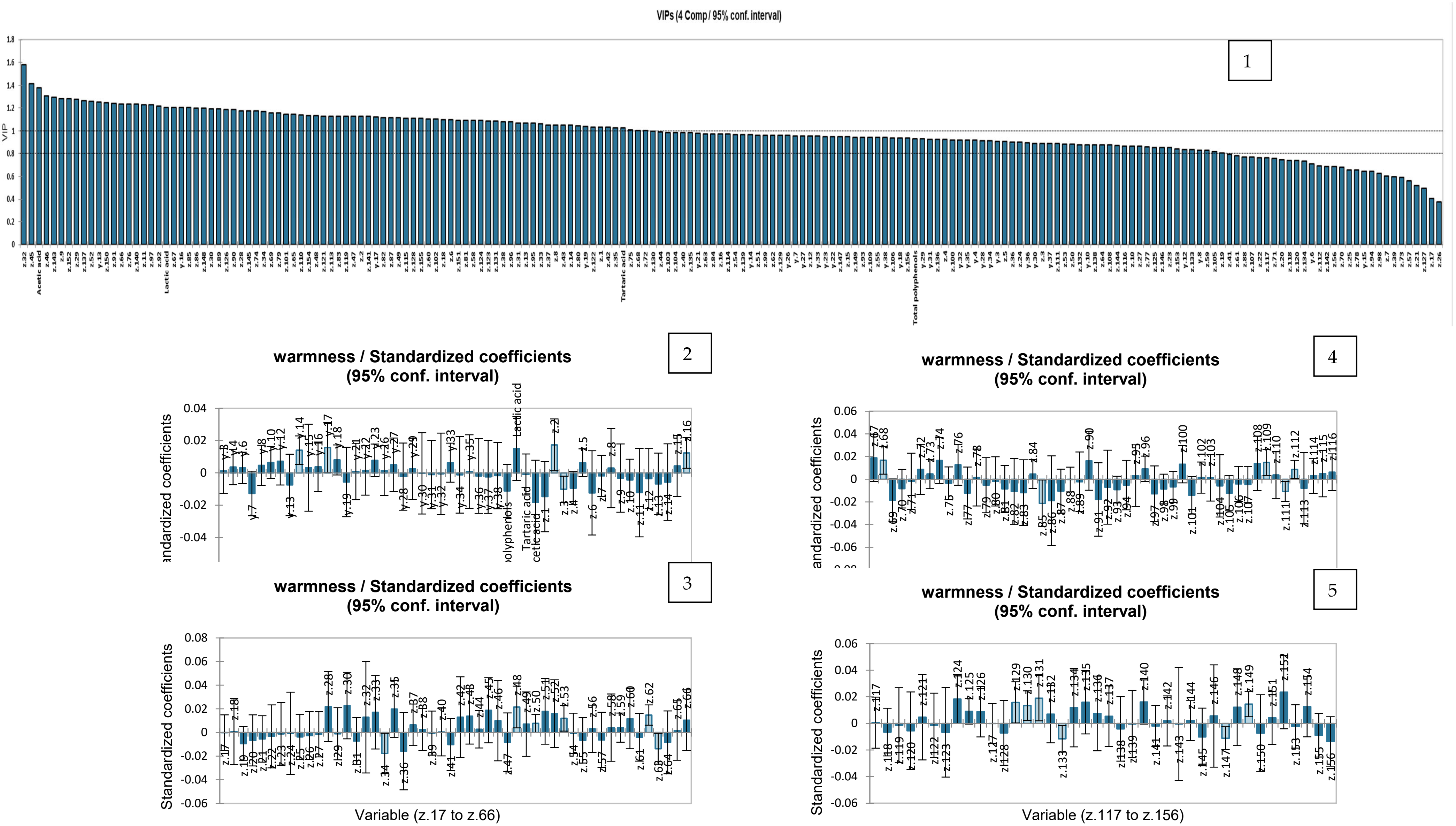
VIPs (4 Comp / 95% conf. interval)
1
VIP
warmness / Standardized coefficients
(95% conf. interval)
2
3
4
5
Standardized coefficients
Variable (z.17 to z.66)
Variable (z.117 to z.156)

6

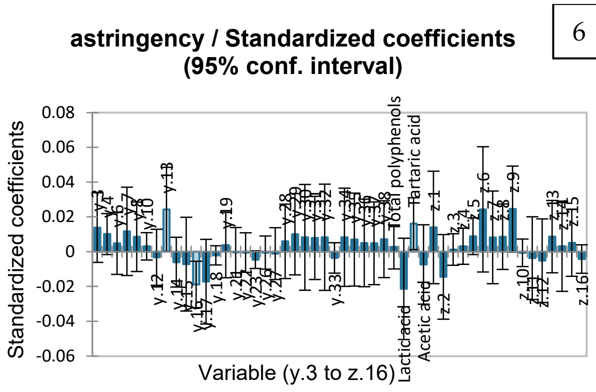

7

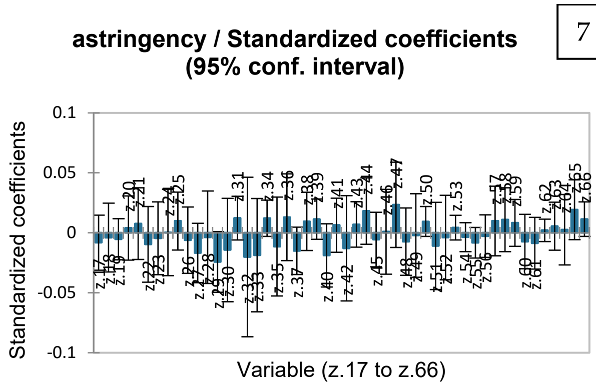

8

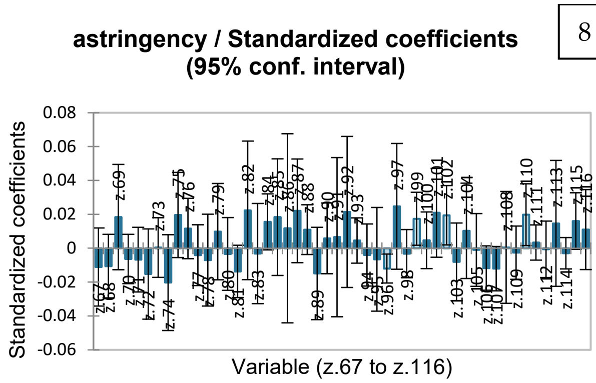

9

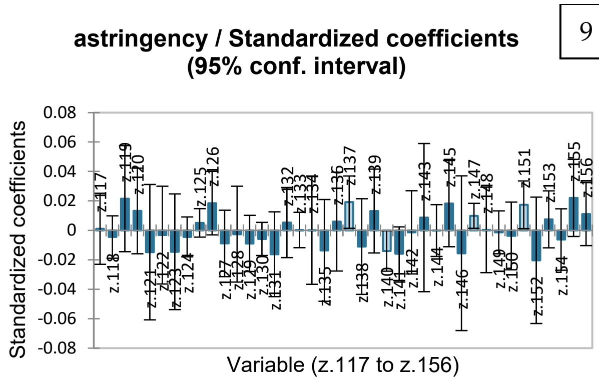

10

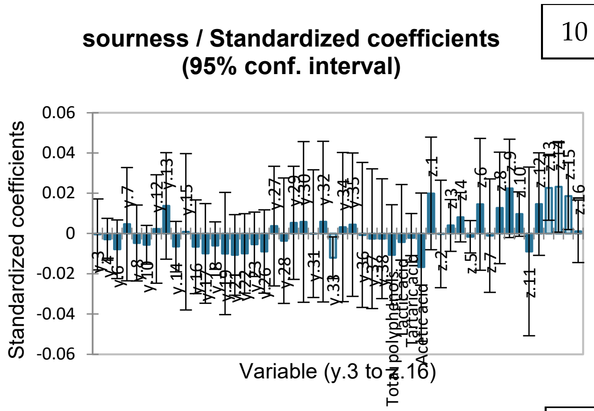

11

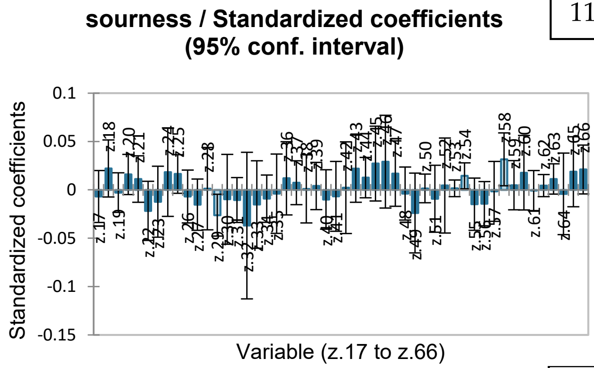

12

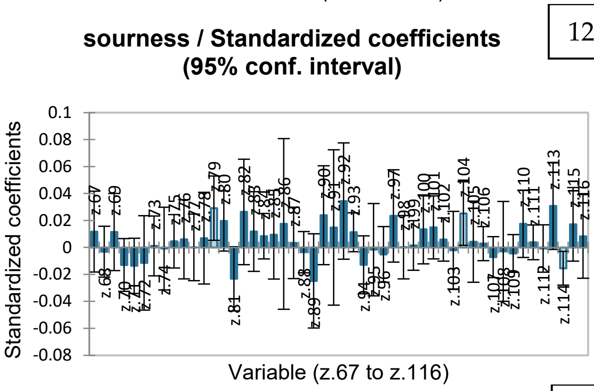

13

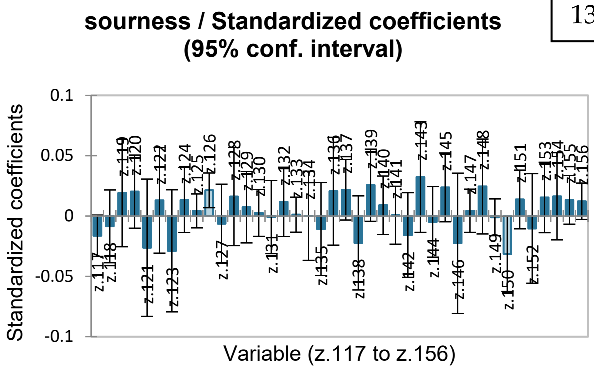

14

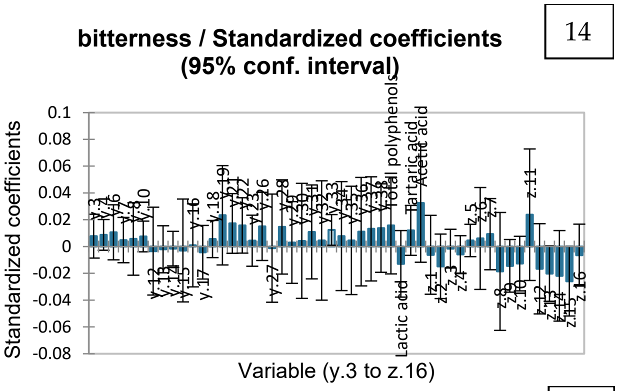

18

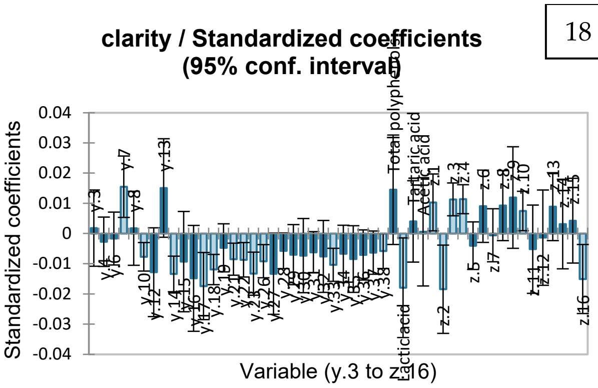

15

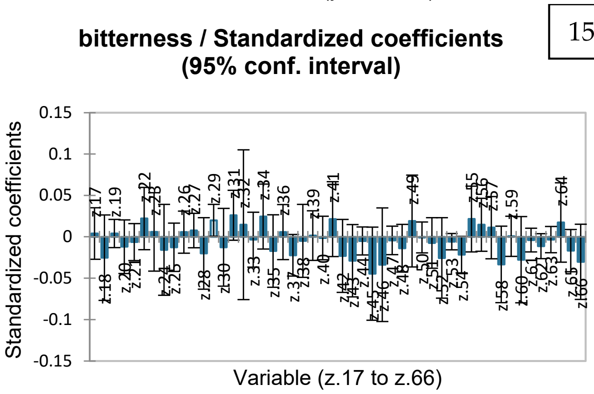

19

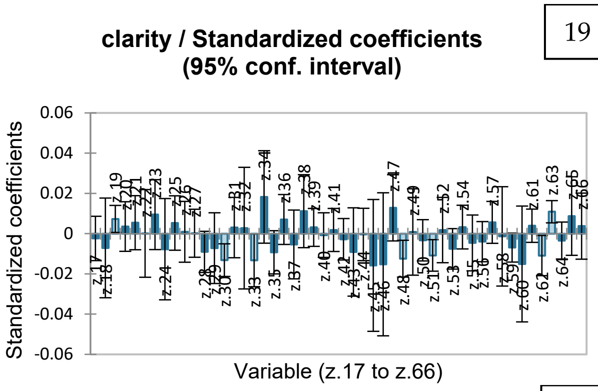

16

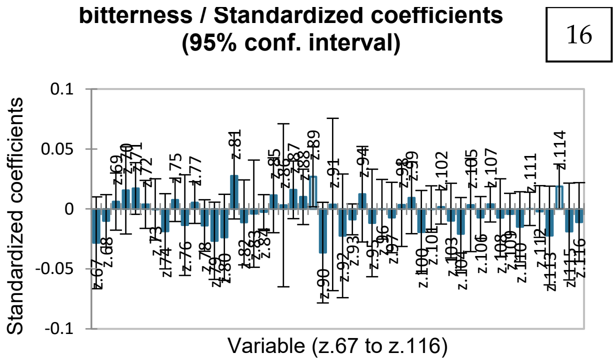

20

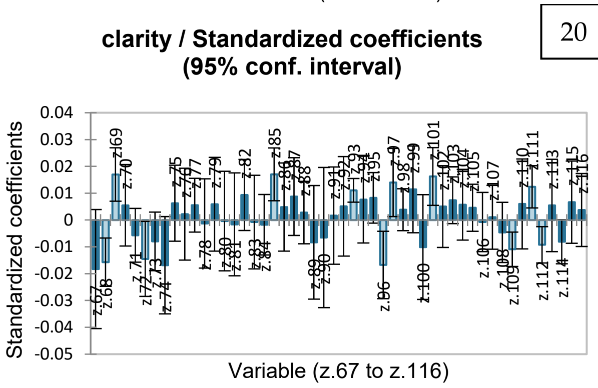

17

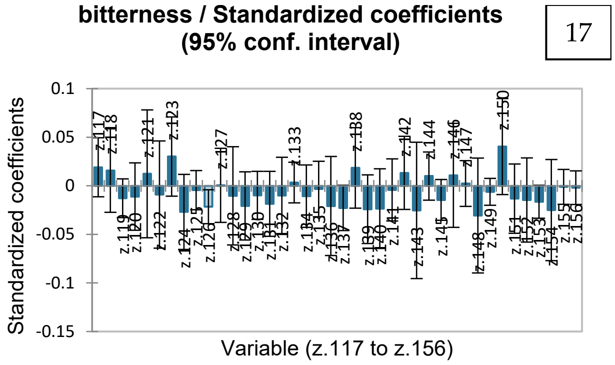

21

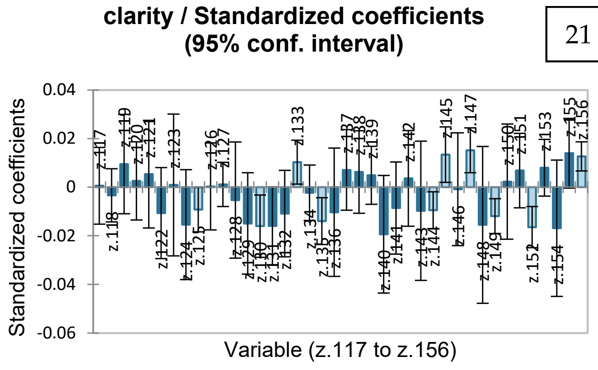

22

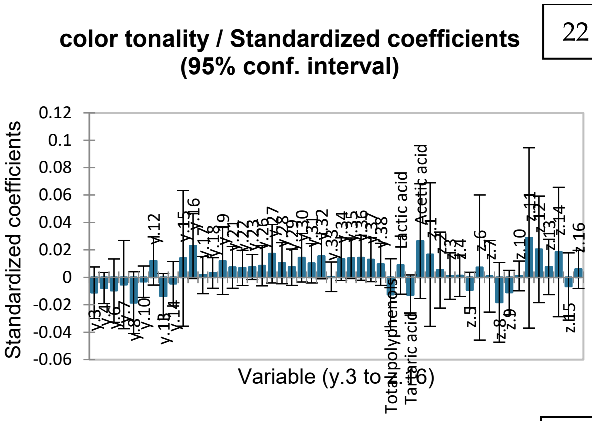

23

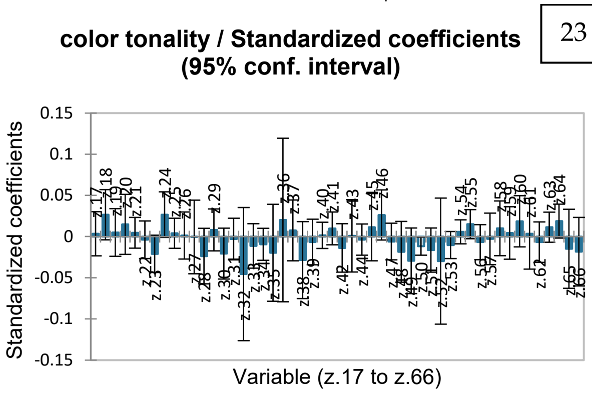

24

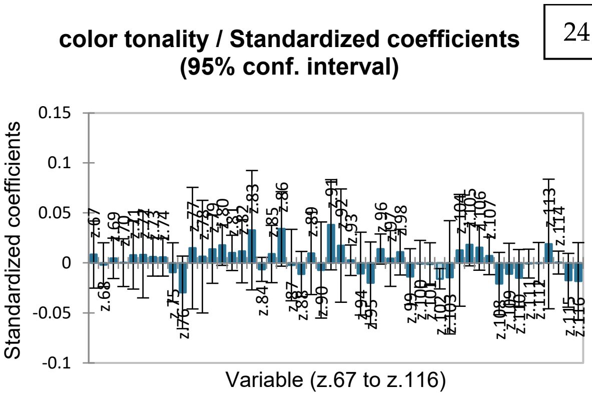

25

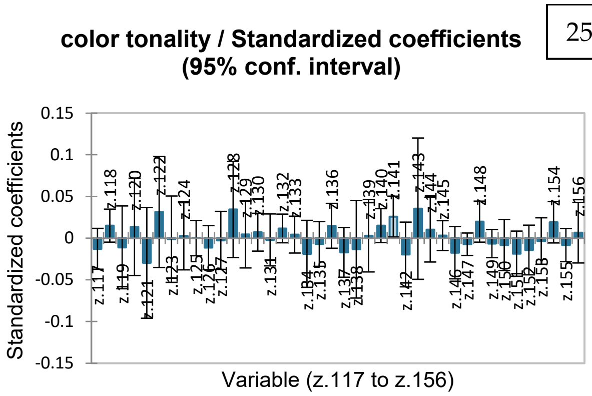

26

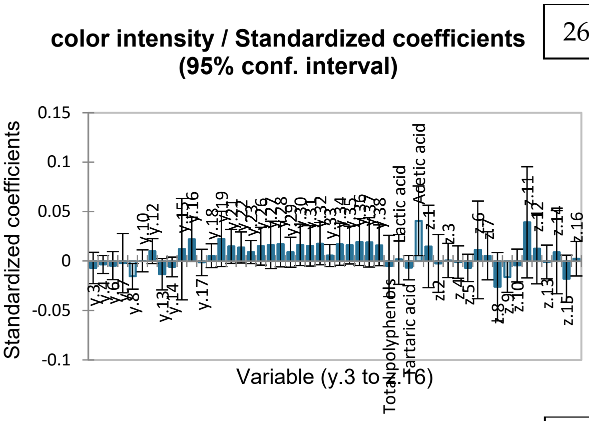

27

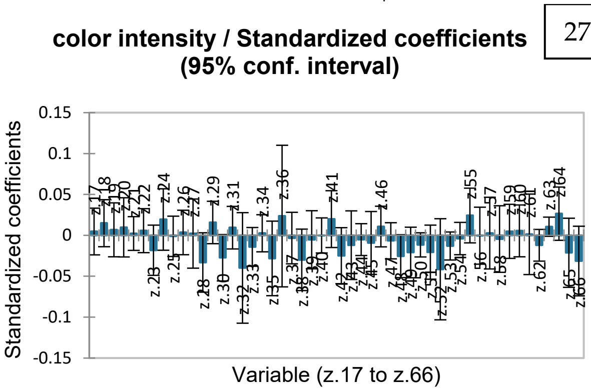

28

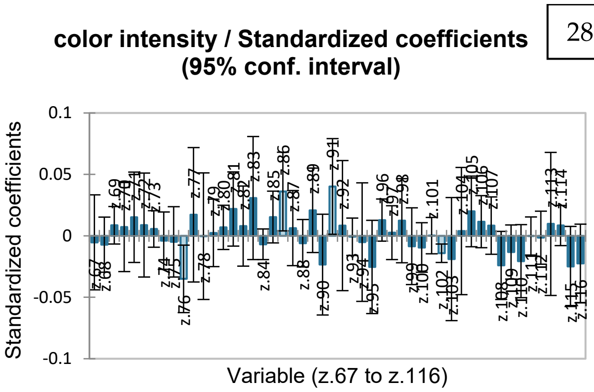

29

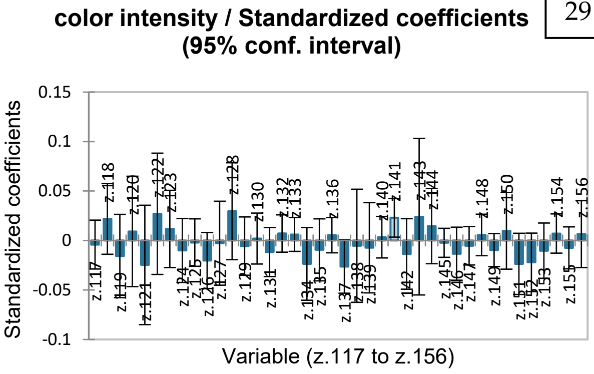

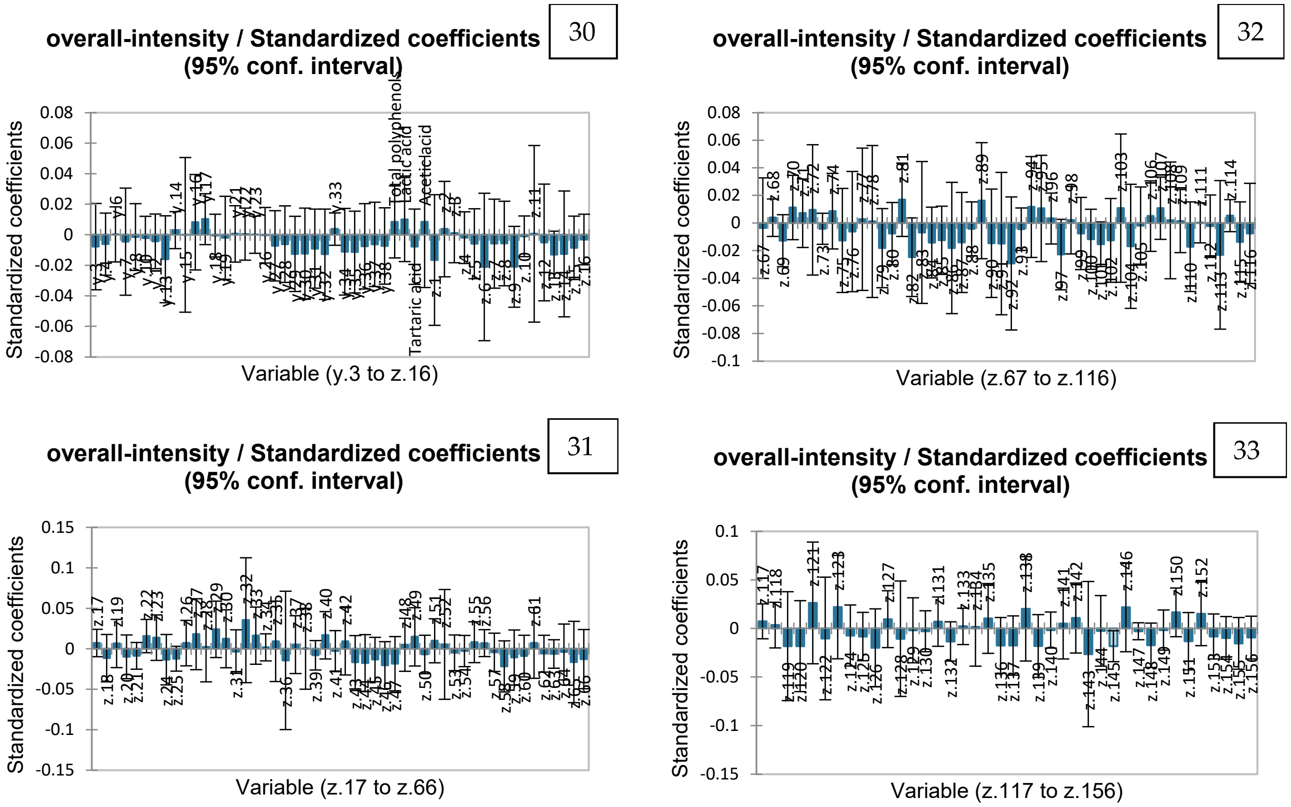

Figure S6. PLS-R for the non-volatile phenolic and anthocyanin compounds, basic oenological parameters with the visual and gustatory data.

Table S2. One way-ANOVA of the oenological parameters. Numbers in bold mean significant differences by Tukey's test ($p<0.05$). A: Mazzon vineyard; C: Aldino vineyard; D: Patone vineyard and E: Eggerhof vineyard.

Vineyard	Malic acid (g/L)	Total polyphenol (mg/L)	Lactic acid (g/L)	Tartaric acid (g/L)	Acetic acid (g/L)
C	0.00 a	1680.50 a	2.17 b	1.18 b	0.34 a
D	0.20 a	1564.50 a	1.79 c	2.04 a	0.23 b
E	0.10 a	1258.50 a	2.67 a	1.11 b	0.27 ab
A	0.01 a	1841.50 a	1.78 c	0.97 b	0.27 ab
Pr > F	0.129	0.285	**0.001**	**0.001**	0.051

Table S3 represents the One-way ANOVA of significant differences by Tukey's test ($p < 0.05$) of anthocyanins in wine samples. Mazzon vineyard (A), Aldino vineyard (C), Patone vineyard (D) and Eggerhof vineyard (E). Only the significant compounds are presented and those which could not be identified are represented with their retention time in min (numbers in bold) and their respective code.

Vineyards	Altitude (m.a.s.l.)	Vineyard exposition	Delphinidin 3-glucoside	petunidin-3-glucoside	peonidin-3-glucoside	Malvidin 3-glucoside	Vitisin A	**11.98** [y.22]	**12.23** [y.23]	**12.33** [y.24]	**12.57** [y.25]	**12.74** [y.26]
C	800	South	234240 a	407281 a	514404 b	3378573 b	144423 a	69839 a	39593 a	111745 a	61009 a	168057 a
D	800	East	277378 a	388532 a	658791 a	3523922 ab	78685 c	48393 ab	30343 ab	89304 a	52274 a	120688 b
E	1050-1150	South	177163 ab	331326 ab	345429 c	3737780 a	128610 ab	42742 ab	33766 ab	85909 a	40990 ab	113239 b
A	350	Nort-west	107343 b	189889 b	522037 b	2594939 c	90609 bc	13489 b	9176 b	33097 b	20958 b	46776 c
Pr > F			0.015	0.017	0.002	0.000	0.009	0.036	0.041	0.006	0.016	0.002

Vineyards	Altitude (m.a.s.l.)	Vineyard exposition	**13.06** [y.27]	**13.22** [y.28]	**13.59** [y.31]	**14.06** [y.33]	**14.54** [y.36]	**14.7** [y.37]	**14.82** [y.38]	**14.98** [y.39]	**15.82** [y.43]	**16.29** [y.45]
C	800	South	61573 a	35604 a	58005 a	18275 a	26123 a	20902 a	26509 a	17788 a	17187 a	22334 b
D	800	East	38914 ab	21221 ab	41364 ab	14243 a	14299 ab	10960 ab	19725 ab	9722 ab	9993 ab	24523 ab
E	1050-1150	South	46711 ab	14313 ab	23680 ab	12803 a	10766 ab	7903 ab	12306 ab	7853 ab	9622 ab	26802 ab
A	350	Nort-west	14244 b	4451 b	1826 b	1044 b	2525 b	2352 b	3477 b	2199 b	5143 b	31409 a
Pr > F			0.022	0.022	0.030	0.007	0.047	0.032	0.020	0.033	0.024	0.047

Table S4: One-way ANOVA on the sensory descriptors. Only the attribute with a significant different by Tukey's test ($p < 0.05$) are presented. Mazzon vineyard (A), Aldino vineyard (C), Patone vineyard (D) and Eggerhof vineyard (E).

Vineyard	Altitude	Exposure	Clarity	Olfactory dry fruit	Olfactory undergrowth	Warmness
A	350	North-west	7.786 a	4.143 b	2.500 b	5.929 b
C	800	South	7.071 ab	5.214 ab	3.286 ab	6.214 ab
D	800	East	7.313 ab	5.313 ab	2.813 ab	6.313 ab
E	1050-1150	South	6.583 b	5.500 a	4.083 a	6.750 a
Pr > F			0.016	0.032	0.035	0.041

Table S5 represents the One-way ANOVA of only the significant phenolic by Tukey's test ($p < 0.05$) in wine. A: Mazzon vineyard; C: Aldino vineyard; D: Patone vineyard and E: Eggerhof vineyard. The most intense and significant phenols identified are *p*-coumaroylquinic acid (z.47); glutathionyl caftaric acid (z.114); coutaric acid (z.133) and astilbin (z.151) reported in bold. Meanwhile, low-intensity phenols not yet identified are reported using codes (z) and are not in bold.

vineyards	Altitude	Exposure	z.2	z.3	z.4	z.5	z.6	z.9	z.10	z.13	z.14	z.16	z.19	z.25	z.27
D	800	East	3083 ab	27667 c	487 c	3549 a	15602 a	3370 a	4793 c	4365 b	1328 b	15341 ab	1709 b	2608 a	249 b
E	1050-1150	South	9368 a	29559 c	448 c	1751 b	1766 a	1773 c	5967 b	4283 b	2200 ab	16514 a	2207 ab	2136 ab	1527 ab
C	800	South	3570 ab	52848 b	1785 b	991 c	1914 a	2037 bc	3870 c	3721 b	1998 ab	13244 ab	2531 ab	1333 b	1359 ab
A	350	North-west	925 b	66314 a	3234 a	150 d	7493 a	2476 b	12236 a	6563 a	2508 a	11710 b	3390 a	2596 a	2630 a
Pr > F			0.035	<0.0001	<0.0001	<0.0001	0.046	0.002	<0.0001	0.004	0.048	0.032	0.030	0.028	0.022

vineyards	Altitude	Exposure	z.28	z.29	z.30	z.34	z.37	**z.47**	z.51	z.52	z.54	z.58	z.59	z.63
D	800	East	57417 ab	2072 b	15529 ab	2669 a	3789 b	**2966 a**	34086 ab	35417 a	7257 bc	11192 a	12102 a	716 b
E	1050-1150	South	57776 a	6747 a	19977 a	1190 a	7049 a	**1909 b**	37980 a	36497 a	11906 ab	11131 a	9382 a	690 b
C	800	South	44350 b	7804 a	8549 c	2859 a	6005 ab	**2209 ab**	35791 a	29579 b	4338 c	7589 b	9353 ab	1299 b
A	350	North-west	44889 ab	5925 a	11330 bc	2933 a	7038 a	**2764 ab**	30287 b	40155 a	14730 a	10645 a	5810 b	3261 a
Pr > F			0.022	0.008	0.006	0.048	0.027	**0.024**	0.011	0.007	0.003	0.015	0.009	0.003

vineyards	Altitude	Exposure	z.69	z.70	z.74	z.76	z.82	z.83	z.84	z.85	z.87	z.91	z.93	z.94
D	800	East	14139 a	367 b	263 b	4816 a	2597 a	1750 b	26492 a	6763 a	3071 a	1345 b	658 b	4236 b
E	1050-1150	South	9902 b	1980 b	4660 a	1932 b	1246 ab	6384 ab	15183 b	2103 b	1024 b	1761 ab	94 b	4225 b
C	800	South	14237 a	11558 a	856 b	986 b	789 b	3456 ab	15497 b	8060 a	1416 b	2841 a	1504 ab	7354 a
A	350	North-west	14998 a	6276 ab	372 b	1941 b	1426 ab	6818 a	8601 c	8188 a	1100 b	1599 ab	2608 a	5471 ab
Pr > F			0.000	0.006	0.003	0.003	0.028	0.039	0.001	0.001	0.013	0.042	0.014	0.030

vineyards	Altitude	Exposure	z.96	z.97	z.98	z.101	z.102	z.105	z.106	z.109	z.110	**z.114**	z.115
D	800	East	4053 b	11836 a	1431 d	12854 a	7480 a	19524 b	720 b	4429 a	6845 a	**11637 b**	7186 a
E	1050-1150	South	6181 a	2056 b	8847 c	8686 b	2958 b	24336 b	2839 a	4464 a	4003 b	**10524 b**	2883 b
C	800	South	5669 a	8985 a	37926 a	10969 ab	2407 b	44980 a	1713 ab	2485 a	4945 ab	**15399 a**	3508 b
A	350	North-west	2587 b	8670 a	17629 b	12358 a	3211 ab	31403 ab	3448 a	2244 a	4326 ab	**2937 c**	3790 b
Pr > F			0.003	0.003	<0.0001	0.009	0.027	0.009	0.014	0.037	0.046	**0.000**	0.003

vineyards	Altitude	Exposure	z.118	z.125	z.130	**z.133**	z.140	z.144	z.145	z.149	**z.151**	z.152	z.153	z.156
D	800	East	227 c	9046 a	134688 ab	**6213 b**	3801 ab	3182 ab	4359 ab	3021 a	**15145 a**	2234 ab	423 b	5703 b
E	1050-1150	South	1175 b	8235 a	152101 a	**6500 ab**	8322 a	3030 ab	1266 b	1618 b	**2452 c**	4495 a	424 b	3561 c
C	800	South	5662 a	5981 b	132870 ab	**9369 a**	4579 ab	3603 a	3452 ab	1631 b	**8368 b**	1854 b	778 a	3795 c
A	350	North-west	207 c	5447 b	105502 b	**8915 ab**	3386 b	1620 b	5959 a	202 c	**4565 c**	1858 b	934 a	8209 a
Pr > F			<0.0001	0.001	0.019	**0.028**	0.038	0.026	0.016	<0.0001	**<0.0001**	0.037	0.008	0.000

Table S6. One way-ANOVA of the oenological parameters. Numbers in bold mean significant different by Tukey's test ($p<0.05$). A_C = no treatment with chitosan; A_CC_C = treatment with chitosan only before harvest; A_CC_CC = treatment with chitosan all year.

Chitosan treatment	Malic acid (± 0.01 g/L)	Total polyphenol (± 0.01 mg/L)	Lactic acid (± 0.01 g/L)	Tartaric acid (± 0.01 g/L)	Acetic acid (± 0.01 g/L)
A_CC_CC	0.000 a	1825.500 a	1.974 a	1.163 a	0.226 b
A_C	0.008 a	1841.500 a	1.784 a	0.969 b	0.275 ab
A_CC_C	0.000 a	1734.000 a	1.895 a	0.996 b	0.323 a
Pr > F	0.465	0.954	0.257	**0.001**	**0.019**

Table S7 represents the One-way ANOVA of significant differences by Tukey's test ($p < 0.05$) of anthocyanins in wines from Mazzon vineyard. A_C = no treatment with chitosan; A_CC_C = treatment with chitosan only before harvest; A_CC_CC = treatment with chitosan all year. Only the significant compounds are presented and those which could not be identified are represented with their retention time (Rt) in min and their respective code.

Mazzon	Delphinidin 3-glucoside	Rt. 6.47 code [y.6]	Rt. 6.66 code [y.7]	Rt. 6.92 code [y.8]	petunidin-3-glucoside	peonidin-3-glucoside	Rt. 14.82 code [y.38]	Rt. 15.82 code [y.43]	Rt. 16.05 code [y.44]	Rt. 16.42 code [y.46]
A_CC_C	101615 b	5972 b	10869 b	21849 b	182705 b	516186 b	3742 a	5456 a	3335 b	2445 b
A_C	107343 b	5779 b	10738 b	21807 b	189889 b	522037 b	3477 a	5143 a	3389 b	2600 b
A_CC_CC	133303 a	6508 a	11444 a	29007 a	220558 a	644495 a	2702 b	3929 b	4387 a	3378 a
Pr > F	0.009	0.024	0.012	0.000	0.023	0.003	0.038	0.017	0.011	0.015

Table S8 represents the One-way ANOVA of only the significant phenolic compounds by Tukey's test ($p < 0.05$) in wine from Mazzon vineyard. A_C = no treatment with chitosan; A_CC_C = treatment with chitosan only before harvest; A_CC_CC = treatment with chitosan all year. The most intense and significant phenols identified are trans-caftaric acid (z.117) and catechin (z.134) reported in bold. Meanwhile, low-intensity phenols not yet identified are reported using codes (z) and are not in bold.

Mazzon	z.9	z.13	z.28	z.29	z.35	z.40	z.42	z.54	z.69	z.84	z.88	z.93	z.95
A_CC_CC	3433 a	8018 a	52844 a	2582 b	3504 a	9997 b	13704 a	16254 ab	16087 a	14414 a	2099 a	3263 a	4986 a
A_CC_C	2584 b	7614 a	40880 b	5612 a	2463 b	12633 a	9375 b	16973 a	16529 a	8207 b	1952 a	2732 b	3729 b
A_C	2476 b	6563 b	44889 b	5925 a	3042 a	12816 a	9113 b	14730 b	14998 b	8601 b	1158 b	2608 b	4562 a
Pr > F	0.027	0.042	0.022	0.022	0.016	0.009	0.017	0.048	0.016	0.021	0.040	0.018	0.027

Mazzon	z.102	z.104	z.108	**z.117**	z.118	z.125	z.126	z.129	**z.134**	z.149
A_CC_CC	4207 a	6134 a	2325 a	**4730 a**	1042 a	6014 a	7093 a	3478 a	**7271 a**	983 a
A_CC_C	2592. b	1662 b	1529 b	**2751 b**	604 b	6086 a	4618 b	2636 b	**4369 b**	92 b
A_C	3211 b	707 b	1557 b	**2907 b**	207 c	5447 b	5340 b	3514 a	**4483 b**	202 b
Pr > F	0.012	0.034	0.047	**0.006**	0.000	0.049	0.009	0.008	**0.000**	0.001
